# Targeting a non-oncogene addiction to the ATR/CHK1 axis for the treatment of small cell lung cancer

**DOI:** 10.1038/s41598-017-15840-5

**Published:** 2017-11-14

**Authors:** Fabian Doerr, Julie George, Anna Schmitt, Filippo Beleggia, Tim Rehkämper, Sarah Hermann, Vonn Walter, Jean-Philip Weber, Roman K. Thomas, Maike Wittersheim, Reinhard Büttner, Thorsten Persigehl, H. Christian Reinhardt

**Affiliations:** 1 Department I of Internal Medicine, University Hospital of Cologne, Cologne, Germany; 2 Cologne Excellence Cluster on Cellular Stress Response in Aging-Associated Diseases, University of Cologne, Cologne, Germany; 3 Department of Cardiothoracic Surgery, University Hospital of Cologne, Cologne, Germany; 4 Department of Translational Genomics, Center of Integrated Oncology Cologne-Bonn, Medical Faculty, University of Cologne, Cologne, Germany; 5 Department of Public Health Sciences, Penn State Milton S. Hershey Medical Center, Hershey, PA USA; 6 Lineberger Comprehensive Cancer Center, University of North Carolina at Chapel Hill, Chapel Hill, NC USA; 7 Department of Radiology, University Hospital of Cologne, Cologne, Germany; 8 Institute for Pathology, University Hospital of Cologne, Cologne, Germany; 9 German Cancer Research Center, German Cancer Consortium (DKTK), Heidelberg, Germany

## Abstract

Small cell lung cancer (SCLC) is a difficult to treat subtype of lung cancer. One of the hallmarks of SCLC is its almost uniform chemotherapy sensitivity. However, chemotherapy response is typically transient and patients frequently succumb to SCLC within a year following diagnosis. We performed a transcriptome analysis of the major human lung cancer entities. We show a significant overexpression of genes involved in the DNA damage response, specifically in SCLC. Particularly *CHEK1*, which encodes for the cell cycle checkpoint kinase CHK1, is significantly overexpressed in SCLC, compared to lung adenocarcinoma. In line with uncontrolled cell cycle progression in SCLC, we find that CDC25A, B and C mRNAs are expressed at significantly higher levels in SCLC, compared to lung adenocarcinoma. We next profiled the efficacy of compounds targeting CHK1 and ATR. Both, ATR- and CHK1 inhibitors induce genotoxic damage and apoptosis in human and murine SCLC cell lines, but not in lung adenocarcinoma cells. We further demonstrate that murine SCLC tumors were highly sensitive to ATR- and CHK1 inhibitors, while *Kras* ^*G12D*^-driven murine lung adenocarcinomas were resistant against these compounds and displayed continued growth under therapy. Altogether, our data indicate that SCLC displays an actionable dependence on ATR/CHK1-mediated cell cycle checkpoints.

## Introduction

Small cell lung cancer (SCLC) is a neuroendocrine lung cancer subtype that is characterized by small cells that express neuroendocrine markers, such as chromogranin or synaptophysin^1^. Further features of SCLC are its extraordinary aggressiveness and tendency to metastasize early^1^, the almost universal sensitivity to genotoxic combination chemotherapy, as well as the typically transient and short duration of the resulting response, leading to the early death of affected patients^1^. Despite recent exome and genome sequencing efforts, no recurrent actionable genomic aberrations have been identified in SCLC, thus far^2,3^. However, the study of SCLC genomes has revealed a number of recurrent aberrations, including an obligate bi-allelic inactivation of *TP53* and *RB1*, frequent losses on the short arm of chromosome 3 and the recurrent inactivation of the histone acetyl transferase genes, *CREBBP* and *EP300* ^3^. In the absence of effective targeted agents, chemotherapy, typically involving cisplatin and etoposide, as well as whole brain irradiation, remain the main therapeutic principles for extensive disease^4^. In addition to chemotherapy, early clinical data suggest that targeting DLL3, a ligand involved in Notch signaling, is clinically active^5,6^. Furthermore, immunotherapy has similarly elicited pronounced and durable responses in SCLC, likely due to the presence of a high mutational burden, which is known to correlate with immunotherapy response^7^.

The combined loss of both *TP53* and *RB1* may lead to massively destabilized cell cycle checkpoints in SCLC cells. In response to genotoxic stress, p53, encoded by *TP53*, undergoes extensive posttranslational modifications, leading to protein stabilization and nuclear translocation, where p53 acts as a transcription factor^8,9^. Among the target genes that are trans-activated by p53 are the cell cycle-regulating genes *CDKN1A*, *14-3-3σ*, *GADD45α*, *RPRM* and others^9^. Thus, it is conceivable that loss of *TP53* is associated with defective cell cycle control in response to endogenous and exogenous genotoxic damage. Moreover, loss of *RB1*, which belongs to a family of genes encoding the so-called pocket proteins RB1, p107 and p130, is associated with uncontrolled cell cycle progression, particularly at the G_1_/S border^10–12^. In early G_1_, RB1 governs the restriction point by sequestering the E2F1 transcription factor^10–12^. Upon phosphorylation by CDK4 and 6, RB1 undergoes a conformational change, leading to the release of E2F1, which subsequently drives the expression of cyclin E, which further enhances CDK activity and ultimately drives an irreversible S-phase entry^10–12^. Thus, loss of *RB1* is thought to lead to a destabilized G_1_/S border.

In addition to p53- and RB1-controlled transcription-mediated cell cycle control, a kinase based cell cycle checkpoint network exists that, when activated by genotoxic damage, leads to a rapid block in cell cycle progression and the subsequent repair of DNA damage. This signaling network is commonly referred to as the DNA damage response (DDR)^13^. The DDR consists of a series of proximal kinases, including ATM, ATR and DNA-PKcs^14,15^. Particularly, ATM and ATR relay their signaling activity through the downstream effector kinases CHK2 and CHK1, respectively^14,15^. We and others recently identified a third branch of cell cycle checkpoint signaling, involving a kinase pathway in which ATM leads to the activation of TAO1, which in turn activates the p38MAPK/MAPKAP-K2 stress kinase complex^16–20^. The three cell cycle checkpoint effector kinases CHK1, CHK2 and MK2 share substrate motif homology, selecting for amino acid sequences with basophilic residues in the Ser/Thr −3 position and hydrophobic residues in the Ser/Thr −5 and +1 position^14,15^. One of the most prominent substrates of these checkpoint effector kinases is the CDC25 family of phosphatases, which are inactivated by CHK1/CHK2/MK2-mediated phosphorylation^14,15^. CDC25 phosphatases mediate de-phosphorylation and subsequent activation of cyclin dependent kinases (CDKs), which are critical drivers of the mammalian cell cycle^21,22^. Thus, DDR-mediated inhibition of CDC25 activity leads to a cell cycle arrest, due to inadequate CDK activity^21,22^.

Here, we show that *CHEK1* mRNA is significantly overexpressed in primary human SCLC, compared to non-small cell lung cancer (NSCLC) samples. We further show that not only CHK1 inhibition, but also ATR inhibition leads to the induction of genotoxic stress and subsequent apoptosis, specifically in SCLC cells, while NSCLC cells display resistance against ATR/CHK1 inhibition. We confirm these results in autochthonous and transplanted murine models of SCLC and NSCLC (both *Tp53*-deficient), as well as xenograft experiments. Overall, our data further implicate inhibition of the ATR/CHK1 cell cycle kinase axis as a potential drug target for the treatment of human SCLC patients. These data are in accordance with previously published observations reporting an increased expression of numerous components of the DDR network, including ATR and CHK1, both on the mRNA and protein level in SCLC, compared to NSCLC^23,24^. Moreover, a recent study demonstrated that the second-generation CHK1 inhibitor prexasertib exerts anti-tumor activity against SCLC *in vitro* and *in vivo* ^24^. Furthermore, prexasertib enhanced the effects of cisplatin and the PARP inhibitor olaparib^24^. Employing proteomic technologies, the group showed that CHK1 and MYC were predictive biomarkers of prexasertib sensitivity^24^.

## Results

### Transcriptome analysis in human lung cancer specimens reveals a significantly increased expression of DNA damage response genes in SCLC, compared to NSCLC

**Figure 1:**
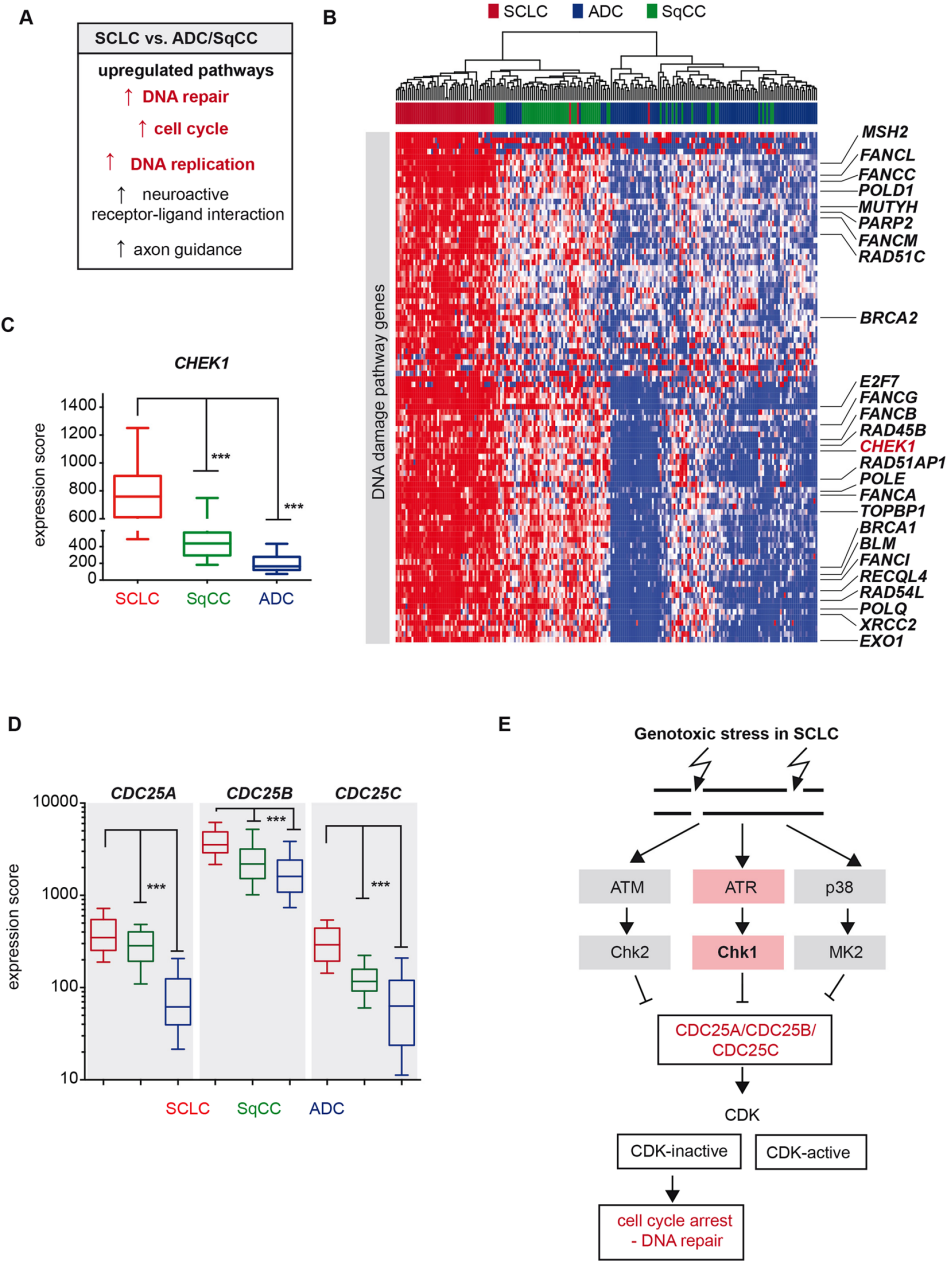
*CHEK1* expression in SCLC. (**A**) Cellular and biological pathways, which are significantly up-regulated in SCLC, compared to lung adenocarcinomas and squamous cell carcinomas. (**B**) Expression profiles of DDR related genes in SCLC and other lung cancer subtypes is represented as a heatmap with red and blue indicating high and low expression, respectively. Tumor samples are arranged from the left to right and sorted according to their expression values. The histological annotation of the lung tumor samples is provided in the color panel above. (**C**) *CHEK1* expression is displayed as a box plot. Whiskers indicate the 10–90 percentile. *** < 0.0001 (Mann Whitney test). (**D**) *CDC25A*, *CDC25B* and *CDC25C* expression is displayed as a box plot. Whiskers indicate the 10–90 percentile. *** < 0.0001 (Mann Whitney test). The histological annotation of the lung tumor samples is provided in the color panel below. (**E**) Simplified schematic representation of kinase-mediated cell cycle checkpoint signaling.

In light of the high proliferation rate of SCLC tumors and the almost universal bi-allelic loss of both tumor suppressor genes, *TP53* and *RB1* ^2,3^, we sought to further characterize the transcriptional profile of SCLC in comparison to other major lung cancer subtypes. We therefore performed a comparative analysis with the transcriptome sequencing data of 214 patient-derived lung tumors^2,3,25–28^. In comparison to lung adenocarcinomas (ADC) and squamous cell carcinomas (SqCC), where co-occurring somatic alterations of *TP53* and *RB1* are less frequent and rather rare^25,26^, SCLC tumors exhibited significantly higher expression levels of genes controlling cell cycle regulation and DNA replication, as well as pathways that emphasize the neuroendocrine features of this lung cancer subtype (Fig. 1A). We furthermore observed a massive up-regulation of mRNAs encoding for different DNA damage response (DDR) and DNA repair pathways (Figs 1A,B, S1), which was similarly observed through previous proteomic studies in SCLC, as well as in a recent transcriptome analysis^23,24^. The detailed analysis of the genes involved in these cellular mechanisms pointed, among others, to *CHEK1* (Fig. 1B). *CHEK1* transcripts were significantly up-regulated in SCLC tumors with a median increase of 2-fold (1.7-fold) and 5-fold (4.6-fold), compared to adenocarcinomas and squamous cell carcinomas, respectively (p < 0.0001, Fig. 1C).

*CHEK1* encodes for one of the three major cell cycle checkpoint effector kinases (CHK1, CHK2, MK2), which in the absence of p53 and RB1 may initiate cell cycle arrest and subsequent DNA repair mechanisms^14,15^. Intriguingly, and in line with poorly controlled cell cycle progression in SCLC, we find that the mRNAs encoding the phosphatases CDC25A, B and C are expressed at significantly higher levels in SCLC, compared to SqCC and ADC samples (Fig. 1D). Together, our observations therefore support the notion that in response to endogenous and exogenous genotoxic stress, SCLC tumors may exploit alternative pathways for DNA repair (Figs 1E, S1) and thus tumor maintenance. The elevated expression levels of *CHEK1* point to a dependence on ATR/CHK1 and may suggest a particularly high vulnerability of SCLC tumors to inhibitors targeting the ATR/CHK1 signaling pathway (Fig. 1E).

### Murine SCLC cell lines display an actionable dependence on the ATR/CHK1 cell cycle checkpoint signaling axis

**Figure 2:**
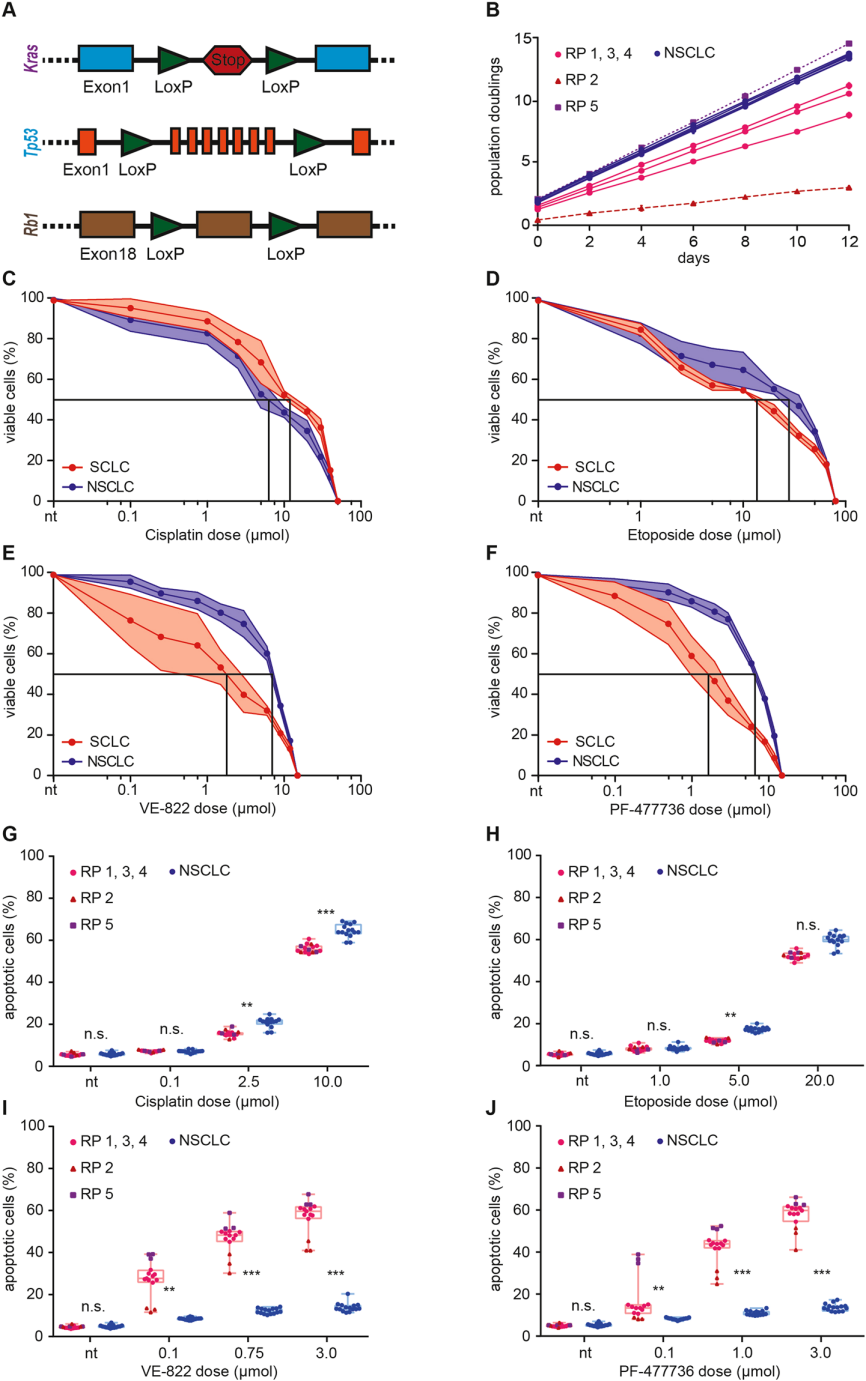
Murine SCLC cell lines are sensitive to ATR- and CHK1 inhibition. (**A**) Schematic representation of the different alleles used in the mouse models: Two groups were designed. *Rb1* ^*fl/fl*^ *;Tp53* ^*fl/fl*^ (RP) and *Kras* ^*LSL.G12D/wt*^ *;Tp53* ^*fl/fl*^ (KP) animals. (**B**) Population doubling time of the five murine RP (SCLC) cell lines and five murine KP (NSCLC) cell lines used in this study. The duration of each passage was 48 hours. KP cell lines displayed rapid proliferation with a minimal variation between the individual cell lines. Proliferation of RP cell lines was more variable. RP1, 3 and 4 displayed comparable proliferation rates. Clone RP2 displayed slow growth kinetics. Clone RP5 showed a high proliferation rate. (**C**–**F**) Intracellular ATP was measured as a surrogate marker for cell viability. Average values of three independent experiments are shown. (**C**) Assessment of cisplatin sensitivity in KP and RP cell lines. KP GI_50_ = 6.7 µMol; RP GI_50_ = 12.3 µMol). (**D**) Assessment of cisplatin sensitivity in KP and RP cell lines. RP GI_50_ = 14.3 µMol; KP GI_50_ = 29.1 µMol. (**E**) RP cell lines were significantly (p < 0.0001) more sensitive to ATR inhibition (VE-822), than KP cell lines. RP GI_50_ = 1.9 µMol; KP GI_50_ = 7.2 µMol. (**F**) RP cell lines were significantly (p < 0.0001) more sensitive to CHK1 inhibition (PF-477736), than KP cell lines. RP GI_50_ = 1.7 µMol; KP GI_50_ = 6.9 µMol. (**G**–**J**) Flow cytometry-based apoptosis measurements. Cells were treated for 48 hours with the indicated drugs at the indicated doses. Apoptosis was assessed by quantification of the Annexin-V/PI double-positive population. Average values of three independent experiments are shown. (**G**–**H**) RP and KP cell lines displayed a similar degree of apoptotic cell death in response to cisplatin and etoposide. (**I**–**J**) Highly significant differences in apoptotic cell death were observed between RP and KP cell lines in response to VE-822 and PF-477736. RP2 was less sensitive to both treatments, compared to RP1, 3 and 4. RP5 displayed the most pronounced sensitivity. Significance was determined using an unpaired t test. Levels of significance were *p < 0.05; **p < 0.01; ***p < 0.001.

With regard to the high expression levels of *CHEK1* in SCLC tumors, we next sought to address the vulnerability of these tumors to inhibitors targeting the CHK1 pathway. In order to directly address this hypothesis, we generated *Rb1* ^*fl/fl*^ *;Tp53* ^*fl/fl*^ mice (Fig. 2A, hereafter referred to as RP) and induced SCLC in these animals through intratracheal application of an adenovirus driving Cre recombinase expression (Ad-CMV-Cre), specifically in pulmonary epithelial cells^29^. In addition, we generated *Kras* ^*LSL.G12D/wt*^ *;Tp53* ^*fl/fI*^ mice (Fig. 2A, hereafter referred to as KP), in which we induced lung adenocarcinomas, through intratracheal instillation of Ad-CMV-Cre^30^. Once mice reached a terminal stage of their disease, animals were sacrificed and we isolated individual tumors to derive stable tumor cell cultures, as previously described^31,32^. We specifically established five SCLC clones and five NSCLC clones. Each individual clone was established from an individual tumor isolated from different mice. As an initial characterization step, we recorded the proliferation rate of the individual cancer cell lines. As shown in Fig. 2B, all five NSCLC clones displayed rapid proliferation with only minimal variation between the individual clones. In contrast, SCLC clones displayed a more divergent proliferation pattern. Clones RP1, 3 and 4 displayed comparable proliferation rates, which were substantially slower than those observed in the NSCLC clones. Clone RP2 displayed substantially slower growth kinetics than RP1, 3 and 4, while clone RP5 showed a proliferation rate that was similar to that observed in the NSCLC clones.

To further characterize the biological properties of the individual cell lines, we next profiled their sensitivity to the frontline chemotherapeutic agents cisplatin (Fig. 2C) and etoposide (Fig. 2D), using intracellular ATP measurements as surrogate markers for cell viability. NSCLC cells were slightly more sensitive to cisplatin (GI_50_ = 6.7 µMol), than SCLC cell lines (GI_50_ = 12.3 µMol). In contrast, NSCLC cells were more resistant to etoposide (GI_50_ = 29.1 µMol), compared to SCLC clones (GI_50_ = 14.3 µMol).

To test the hypothesis that ATR- or CHK1 inhibitors display particular efficacy in SCLC cells, compared to NSCLC cells, we next profiled the sensitivity of our cell line panel to the ATR inhibitor VE-822 and the CHK1 inhibitor PF-477736. Of note, PF-477736 has been extensively profiled in a kinase screening panel representing >100 protein kinases. Among the profiled kinases, only 7 enzymes were inhibited by PF-477736 with <100-fold selectivity, namely VEGFR2, Aurora-A, FGFR3, FLT3, CSF1R, RET and YES^33^. PF-477736 displays a ~100-fold selectivity for CHK1, compared to CHK2^33^. Similarly, VE-822 is a selective ATR inhibitor with IC_50_ values of 0.019 µM, 2.6 µM, and 18.1 µM for ATR, ATM and DNA-PK, respectively^34^. VE-822 displays negligible activity against mTOR and PI3Kγ^34^. Both PF-477736 and VE-822 are currently being investigated in clinical trials (e.g. NCT00437203, NCT02723864). As shown in Fig. 2E, SCLC cells were significantly (P < 0.0001) more sensitive to ATR inhibition through VE-822, than NSCLC cells (GI_50_ = 1.9 µMol vs. GI_50_ = 7.2 µMol, respectively). Similarly, SCLC cells were significantly (P < 0.0001) more sensitive to PF-477736, compared to NSCLC cell lines (GI_50_ = 1.7 µMol vs. GI_50_ = 6.9 µMol, respectively) (Fig. 2F). As ATR and CHK1 operate in a linear signaling pathway, the observation that VE-822 and PF-477736 both display cytotoxic activity in SCLC cells, cross- validates the biological relevance of the ATR/CHK1 pathway for the survival of SCLC cells. To further validate ATR and CHK1 as the relevant drug targets and to exclude structure-specific off-target effects, we re-screened our cell line panel by replacing VE-822 and PF-477736 with alternative ATR- and CHK1 inhibitors that were based on entirely distinct chemical motifs. VE-822 was exchanged against AZD-6738, and PF-477736 was replaced with AZD-7762. As shown in Fig. S2, Both AZD-6738 and AZD-7762 displayed similar cytotoxicity as VE-822 and PF-477736 in SCLC cell lines, while NSCLC cell lines remained largely unaffected, even at a concentration of 3 µM.

As cell viability assessments through intracellular ATP measurements do not distinguish between growth retardation and active cell death, we next performed flow cytometry experiments to directly test the hypothesis that ATR- and CHK1 inhibitors induce apoptotic cell death in SCLC cell lines (Fig. 2G–J). We specifically assessed the percentage of Annexin-V/PI double positive cells in response to increasing doses of cisplatin and etoposide, which served as positive controls for the induction of apoptosis in our cell line panel. While both chemotherapeutic agents induced substantial levels of apoptosis in a dose-dependent manner, we did not detect any substantial differences in the sensitivity of SCLC and NSCLC cell lines to these agents at lower concentrations (Fig. 2G,H). Of note, at higher doses of cisplatin (2.5 µM and 10 µM) and etoposide (5 µM), NSCLC clones displayed a significantly higher degree of apoptosis, than their SCLC counterparts. Furthermore, there were no significant differences in the chemotherapy sensitivity between the different SCLC clones that differed in their individual proliferation rates (cf. Fig. 2B). In full agreement with our cell viability assays (cf. Fig. 2E,F), we observed highly significant differences between SCLC and NSCLC cell lines in response to VE-822 and PF-477736 (Fig. 2I,J, respectively). While NSCLC cells displayed only a mild, dose-dependent increase in the percentage of apoptotic cells, SCLC cells were significantly more sensitive to both agents. Of note, the slow growing cell line RP2 was less sensitive to VE-822 and PF-477736, compared to cell lines RP1, 3 and 4. The rapidly proliferating SCLC cell line RP5 displayed the most pronounced sensitivity to the cell cycle checkpoint abrogating agents VE-822 and PF-477736. Thus, overall, we provide data from two distinct experimental assays to show that murine SCLC cell lines are highly sensitive to ATR- and CHK1 inhibition, compared to murine NSCLC cells, which were largely resistant. Intriguingly, the NSCLC cell lines in our experiments displayed higher proliferation rates, than the SCLC cell lines included in our panel (Fig. 2B). Thus, we observed an entity-specific sensitivity to cell cycle checkpoint abrogation. Furthermore, within the SCLC entity, we could show a proliferation rate-dependent VE-822 and PF-477736 sensitivity.

### Inhibition of ATR and CHK1 induces genotoxic stress in murine SCLC cell lines

**Figure 3:**
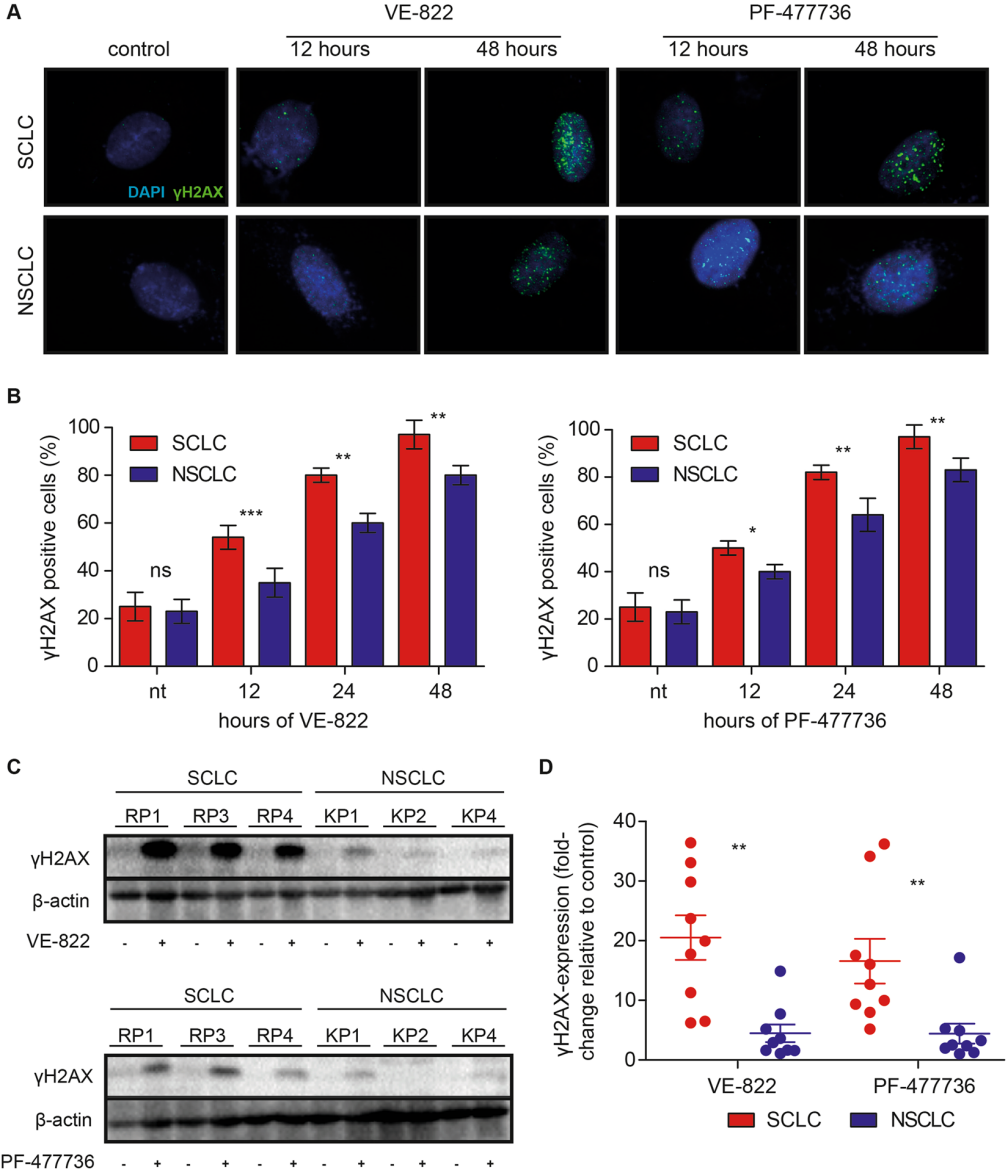
ATR- and CHK1 inhibition induce genotoxic damage in murine SCLC cell lines. (**A** and **B**) KP1–5 and RP1, 3 and 4 cell lines were stained with an antibody detecting γH2AX and a DAPI counterstain. VE-822 and PF-477736 induced genotoxic damage in all RP and in KP cell lines. (**A**) Representative immunofluorescence images are shown. Displayed are images derived from RP1 (SCLC) and KP1 (NSCLC) cell lines that were treated for 12 and 48 hours with VE-822 and PF-477736. The controls were exposed to vehicle solution for 48 hours. (**B**) The percentage of γH2AX-positive cells was quantified. VE-822 (0.75 µM) and PF-477736 (1.0 µM) induced significantly more DNA damage in RP, than in KP cell lines following 12, 24 and 48 hours of drug exposure. (**C-D**) Immunoblot-based assessment of γH2AX induction in KP1, 2, 4 and RP1, 3 and 4 cell lines after a vehicle-, VE-822- (0.75 µM)- or PF-477736 (1.0 µM) treatment (48 hours). β-actin was used as a loading control. (**C**) Representative immunoblot images are shown for the indicated RP- and KP cell lines following the indicated treatment regimens. (**D**) The intensity of the enhanced chemiluminescence signal was quantified, using densitometry. RP cell lines displayed a significantly stronger γH2AX signal, than KP cell lines, following VE-822 and PF-477736 treatment.

We next aimed to further characterize the biological effects of ATR- and CHK1 inhibition in our murine lung cancer cell line panel. We specifically investigated the occurrence of genotoxic lesions in a longitudinal fashion, as ATR and CHK1 kinase activity is critical for a proper progression through S-phase^15,35^. Corroborating this role of the ATR/CHK1 pathway for genome maintenance in S-phase, *Atr* and *Chek1* constitutive knockout mice were shown to be embryonically lethal^36,37^. Furthermore, *Chek1* conditional knockouts display signs of genotoxic damage, upon *Chek1* deletion^38^. Lastly, ATR- and CHK1 inhibitors have previously been shown to cause genotoxic damage^39,40^. To interrogate the effects of ATR- and CHK1 inhibition on genome integrity, we assessed the induction of genotoxic damage, using indirect immunofluorescence. We specifically employed an antibody to detect phospho-Ser-139 in the histone variant H2AX (γH2AX), an established marker for DNA damage^16,32,41,42^. These experiments revealed that VE-822 (0.75 µM) and PF-477736 (1.0 µM) inflicted genotoxic damage in both, NSCLC and SCLC cell lines (Figs 3A,B, S3A–C). In line with a significantly enhanced toxicity, specifically in SCLC cells (cf. Figs 2, S2), VE-822 and PF-477736 induced significantly more DNA damage in SCLC cells, than in NSCLC cell lines at 12, 24 and 48hrs of drug exposure (Figs 3A,B, S3A–C). Of note, even RP2 cells, which displayed the lowest proliferation rate (cf. Fig. 2B), showed VE-822- and PF-477736-induced γH2AX indices that were comparable to those observed in the remaining SCLC cell lines (Fig. S3A–C). To further substantiate these immunofluorescence data, we next performed immunoblot experiments to reproduce our observations in a distinct assay (Figs 3C and D, S3D,E). For this purpose, we treated our cell line panel with VE-822 (0.75 µM, 48 hours) or PF-477736 (1.0 µM, 48 hours). Vehicle-treated cells served as a control. Upon completion of drug exposure, cells were lysed, separated on SDS-PAGE and γH2AX was visualized using a specific antibody. β-actin staining served as a loading control. The intensity of the resulting enhanced chemiluminescence signal was quantified using densitometry. Fully in line with our immunofluorescence data, SCLC cell lines displayed a significantly stronger γH2AX signal following VE-822 and PF-477736 treatment, than their NSCLC counterparts (Figs 3C and D, S3D,E). Thus, both ATR- and CHK1 inhibition induced genotoxic damage that was significantly more pronounced in SCLC, compared to NSCLC cell lines.

### Murine SCLC, but not NSCLC, displays an actionable dependence on the ATR/CHK1 kinase branch, *in vivo*

**Figure 4:**
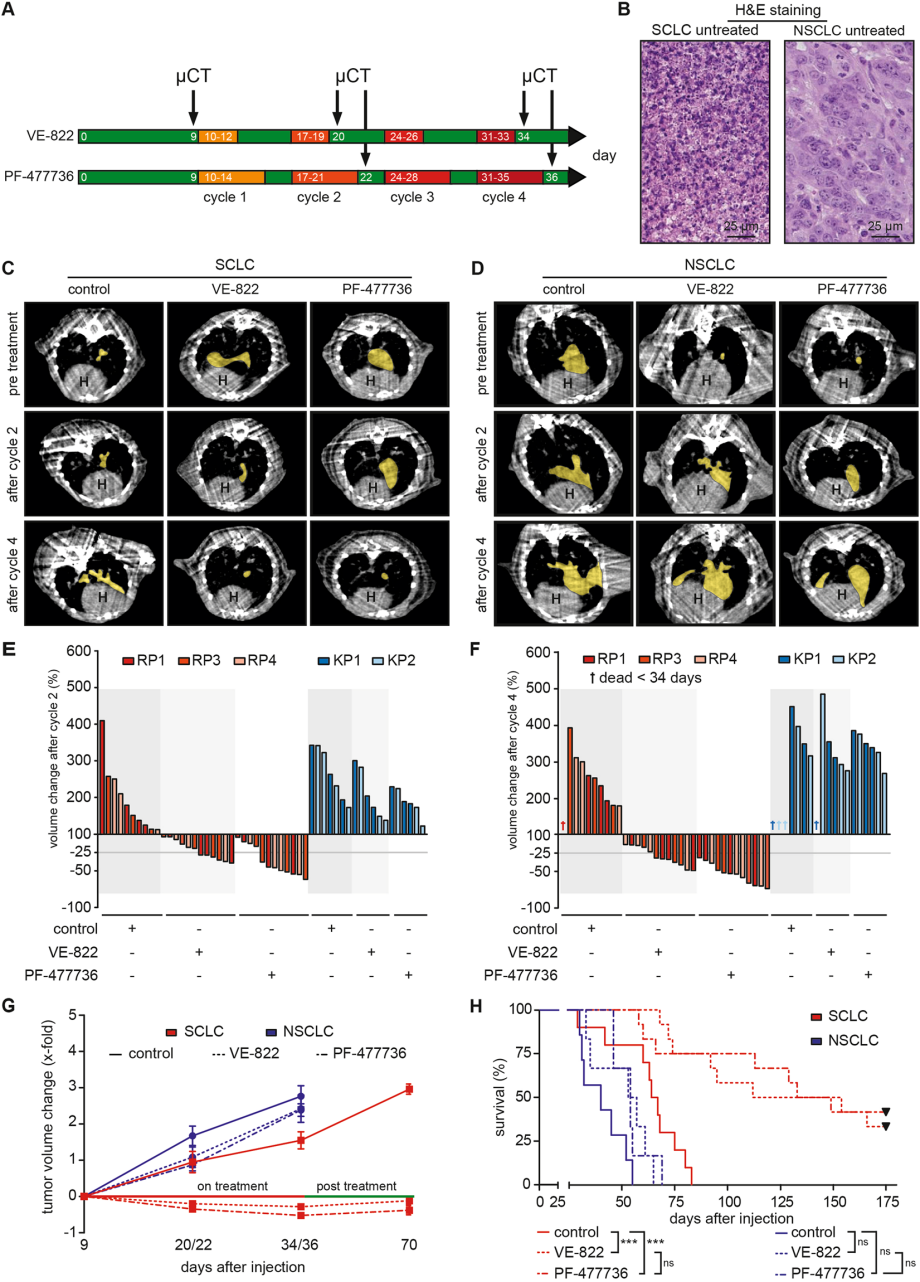
ATR- and CHK1 inhibition displays therapeutic efficacy in SCLC tumors, *in vivo*. (**A**) SCLC and NSCLC tumor formation was assessed by µCT imaging 9 days after intrathoracic injection of RP1, 3, 4 or KP1, 2 cells. For each tumor entity, mice were randomly allocated to a control cohort (not shown in (**A**)) and the indicated treatment cohorts. Four cycles of treatment were applied. The VE-822-treated cohort received treatment weekly for three consecutive days at a dose of 30 mg/kg by oral gavage. The PF-477736-treated cohort received treatment weekly for five consecutive days at a dose of 20 mg/kg by intraperitoneal injection. Tumor volume changes were monitored by µCT imaging at the end of the second and the forth cycle, as indicated. (**B**) Representative images of H&E staining form untreated mice bearing SCLC or NSCLC tumors from the allograft model. Scale bar 25 μm. (**C**–**D**) Tumor volume was assessed by μCT imaging for treated and untreated mice. Imaging revealed SCLC tumor shrinkage in response to VE-822 and PF-477736 treatment after two and four cycles of treatment. Mice bearing NSCLC tumors displayed continued tumor growth in response to both compounds. Representative μCT images of both, SCLC- or NSCLC-bearing mice are shown pre-treatment and after two and four cycles. The yellow area indicates individual tumor lesions. The heart is indicated (**H**). (**E**–**F**) Tumor volume changes in response to the indicated treatments were normalized to baseline tumor volumes, assessed prior to therapy initiation. NSCLC tumors grew faster, than SCLC tumors. Vehicle-treated mice showed continuous tumor growth throughout the observation period. SCLC tumors displayed significant tumor shrinkage after cycle 2 and 4 in response to VE-822 and PF-47736 treatment. NSCLC tumors did not respond to either of the treatments. Three mice from the NSCLC control cohort and one animal from the VE-822-treated NSCLC cohort died before completion of cycle four. (**G**) VE-822- and PF-477736 treatment led to tumor volume reduction in SCLC, but not in NSCLC tumors. Vehicle-treated mice showed continuous tumor growth throughout the observation period. No NSCLC animal survived the post treatment phase until day 70. VE-822- and PF-477736-treated SCLC tumors remained stable after completion of treatment. (**H**) Survival curves of all mice (displayed in Kaplan-Meier format) were calculated from tumor injection up to day 175 and compared by log-rank (Mantle Cox) test. VE-822 and PF-477736 treatment increased overall survival in mice bearing SCLC tumors significantly, compared to the vehicle-treated control group. VE-822- and PF-477736-treated NSCLC-bearing mice showed only a mild survival benefit, compared to their vehicle-treated counterparts.

To further assess the efficacy of ATR- and CHK1 inhibitors *in vivo*, we next performed orthotopic transplantation experiments. For this purpose, we transplanted 2 × 10^6^ SCLC cells (RP1, 3 and 4) and 1.5 × 10^6^ NSCLC cells (KP1 and 2) into the lungs of 8–12-week-old syngeneic *C57BL/6 J* recipient mice. Tumor manifestation was documented through µCT imaging (Fig. 4A).

Of note, we performed hematoxylin/eosin- (Fig. 4B) and Ki-67 stainings (Fig. S4A) of lung tumor sections isolated from transplant recipients, to verify that the transplanted SCLC- and NSCLC-derived cell lines retain their histological properties and proliferation characteristics upon serial passaging *in vitro* and subsequent transplantation.

Once CT-morphologically verified tumors had formed in the recipient animals, mice were treated with four cycles of VE-822 (30 mg/kg, orally, on days 1–3 of a 7-day cycle), PF-477736 (20 mg/kg, i.p., on days 1–5 of a 7-day cycle) or treated with vehicle control. Tumor volume assessments were performed, using µCT imaging on the day prior to cycle 1, as well as after completion of cycle 2 and 4 (Fig. 4A,C–G). When treated with vehicle solution, both SCLC and NSCLC tumors displayed continuous growth throughout the four cycle observation period (Figs 4C–F, S4B,C). Of note, NSCLC tumors grew more rapidly, compared to their SCLC counterparts. Due to this more rapid proliferation pattern, one vehicle-treated animal harboring KP1-derived tumors and two vehicle-treated animals carrying KP2-derived tumors did not survive until the end of cycle four (Figs 4F, S4B). Similarly, NSCLC tumors displayed continued growth when treated with VE-822 or PF-477736 (Figs 4D–F, S4B), essentially mimicking the resistance phenotype that we had observed *in vitro* (Fig. 2C–J). We note that one VE-822-treated animal carrying KP1-derived tumors did not survive until completion of the fourth cycle, due to massive tumor progression (Figs 4F, S4B). In marked contrast and in line with our *in vitro* data, all SCLC tumors displayed significant volume shrinkage in response to VE-822 and PF-47736 compared to vehicle-treated animals, both following cycle 2 (VE-822: p = 0.0004; PF-477736: p < 0.0001) and 4 (VE-822: p = 0.0001; PF-477736: p < 0.0001) (Figs 4C,E–G, S4C). These µCT-morphological tumor response data also translated into highly significant survival gains of VE-822- and PF-477736-treated animals carrying SCLC tumors, compared to their vehicle-treated counterparts (p < 0.0001 and p = 0.0008, respectively) (Fig. 4H). While animals harboring vehicle-treated SCLC tumors displayed a median survival of 66 days following initiation of treatment, VE-822-teated mice showed a median survival of 133 days (Fig. 4H). PF-477736-treated SCLC-bearing animals showed a median survival of 141 days (Fig. 4H). Contrary to these substantial therapeutic effects inflicted by VE-822 and PF-477736 in SCLC-bearing animals, we only observed a minute survival extension in VE-822- and PF-477736-treated NSCLC-bearing mice (Fig. 4H). Median survival in vehicle-treated NSCLC-bearing mice was 40 days, compared to 55 and 54 days following VE-822- and PF-477736-treatment, respectively (Fig. 4H).

**Figure 5:**
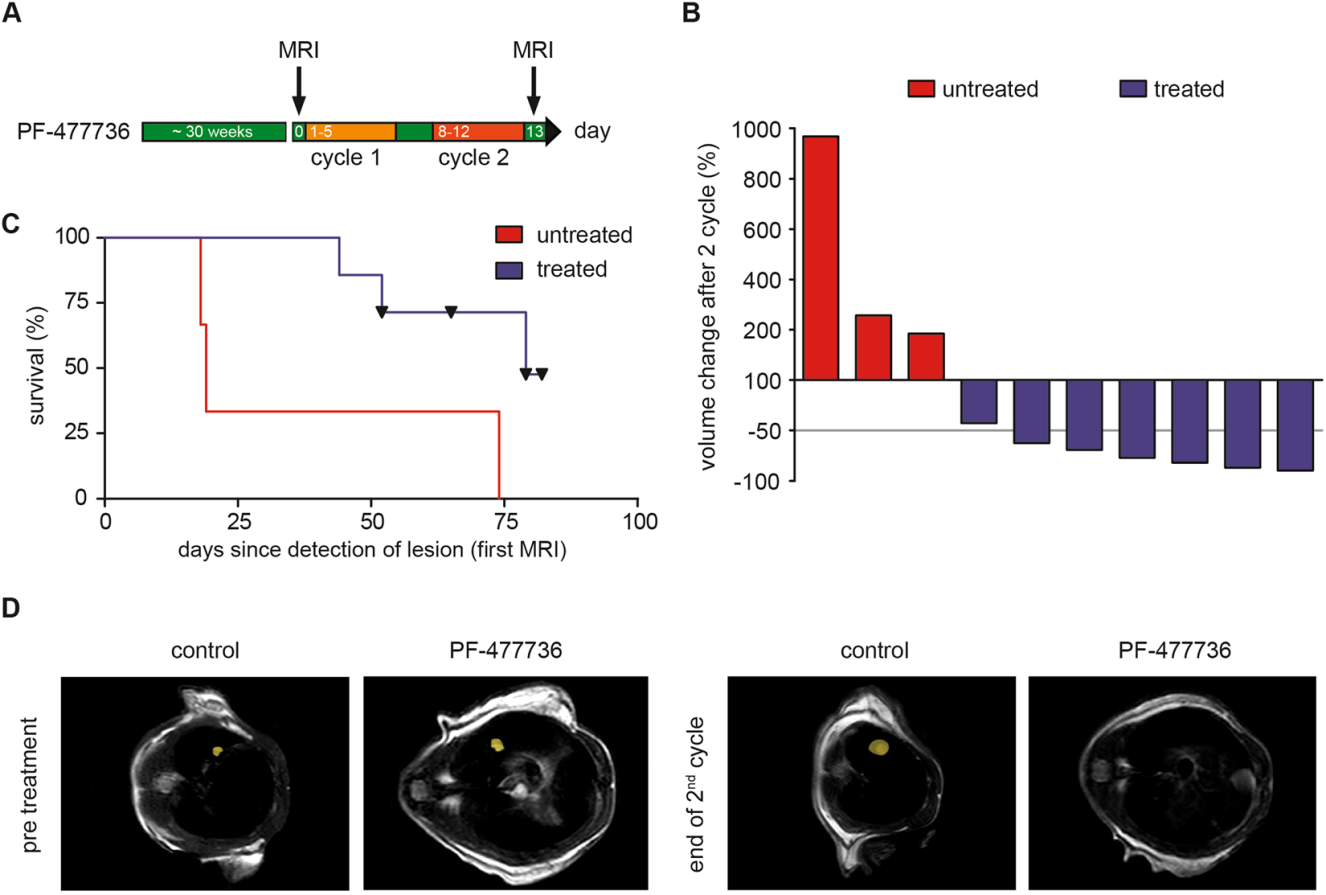
CHK1 inhibition displays therapeutic efficacy in autochthonous SCLC tumors, *in vivo*. (**A**) SCLC tumor formation was assessed by MRT. Mice were randomly allocated to a control cohort (not shown in (**A**)) and the indicated treatment cohort. Two cycles of treatment were applied. The PF-477736-treated cohort received treatment weekly for five consecutive days at a dose of 20 mg/kg by intraperitoneal injection. Tumor volume changes were monitored by MRT imaging at the end of the second cycle, as indicated. (**B**) Tumor volumes were assessed by MRT imaging for PF-477736-treated and vehicle-treated mice. Imaging revealed SCLC tumor shrinkage in response to PF-477736 treatment after two cycles of treatment. Vehicle-treated animals displayed continued tumor growth. Tumor volume changes in response to the indicated treatments were normalized to baseline tumor volumes, assessed prior to therapy initiation. (**C**) Survival curves displayed in Kaplan-Meier format were calculated from initial tumor manifestation and compared by log-rank (Mantle Cox) test. PF-477736 treatment increased overall survival in mice bearing autochthonous SCLC tumors, compared to the vehicle-treated control group. (**D**) Representative MRT images of SCLC-bearing mice are shown pre-treatment and after two cycles of PF-477736 exposure. The yellow area indicates individual tumor lesions.

To further substantiate these observations, we next employed an autochthonous mouse model of SCLC. For that purpose, we induced SCLC in *Tp53* ^*fl/fl*^;*Rb1* ^*fl/fl*^ mice through intratracheal Ad-CMV-Cre instillation, as previously described (Fig. 5)^29^. Tumor onset was monitored by serial MRT imaging. Once MRT-morphologically-verified tumors had formed, we initiated treatment with two cycles of PF-477736 (20 mg/kg, i.p., on days 1–5 of a 7-day cycle), or vehicle control (Fig. 5A). While tumors displayed continuous growth in vehicle-treated animals, all tumors in the PF-477736-exposed cohort displayed a substantial volume reduction after completion of the second cycle (Fig. 5B). This morphological treatment response also translated into a significant survival benefit in the PF-477736-treated cohort, compared to the vehicle-treated animals (median survival 79 days, vs 19 days, respectively, p = 0.0286)(Fig. 5C,D). Collectively, our data derived from transplant models, as well as autochthonous SCLC models, indicate that cell cycle checkpoint inhibition through ATR- and CHK1 inhibition displays selective therapeutic efficacy in murine SCLC tumors, compared to NSCLC tumors, *in vivo*. We note that the adverse effects of VE-822 and PF-477736 were generally mild, *in vivo*. No animal reached the predefined termination criteria (weight loss >10%, signs of infection, bleeding, diarrhea, adynamia or affected social behavior), due to compound toxicity.

### Human SCLC cell lines display high levels of CHK1 mRNA expression, as well as sensitivity to ATR- and CHK1 inhibition in xenograft models

**Figure 6:**
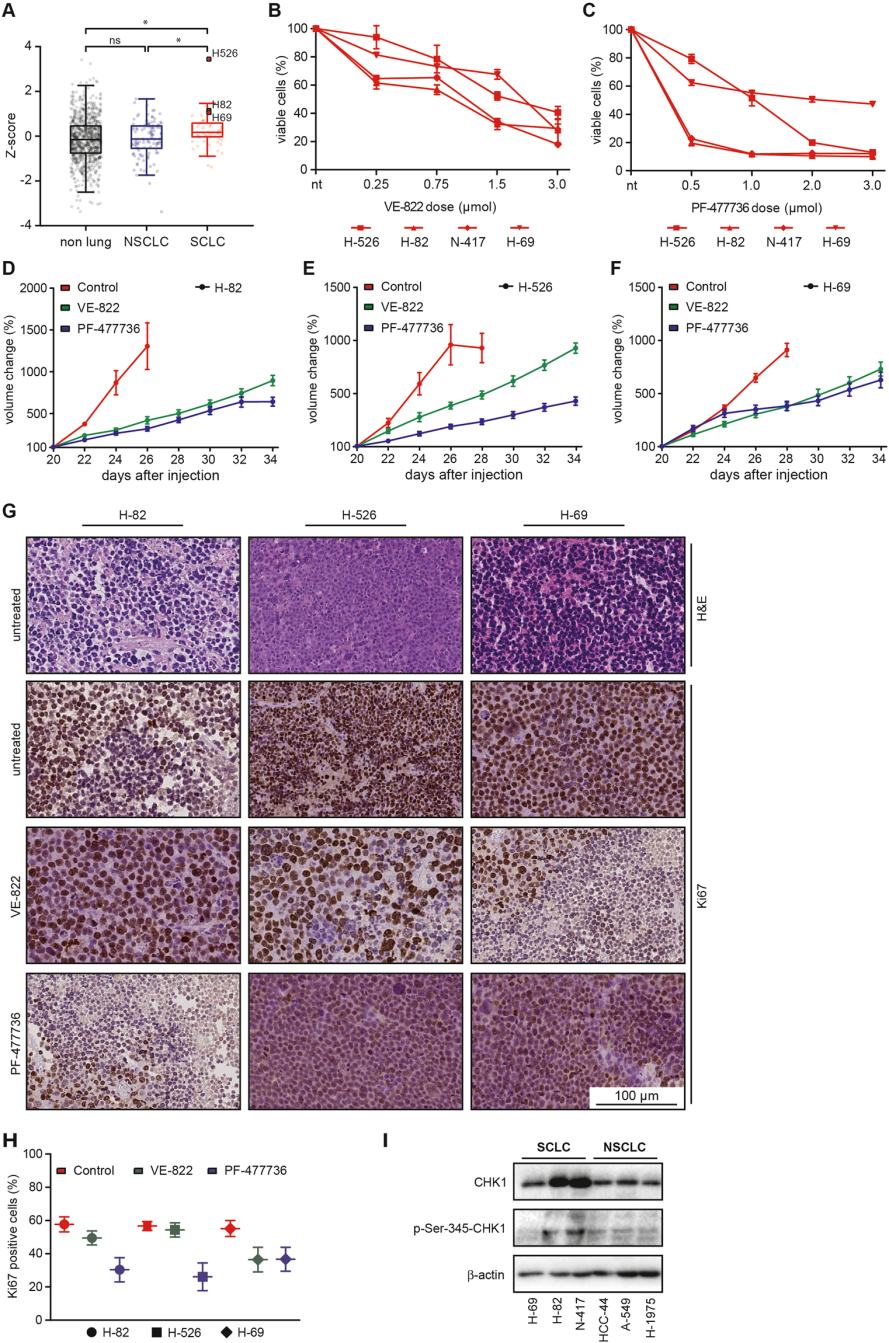
Human SCLC cells are sensitive against ATR- and CHK1 inhibitors in a xenograft model, *in vivo*. (**A**) Displayed are the relative *CHEK1* mRNA expression levels of 970 human cancer cell lines, including 61 SCLC and 109 NSCLC cell lines, as well as 800 non-lung cancer cell lines. (**B–C**) Intracellular ATP levels of four different human SCLC cell lines were measured to assess cell viability. Average values of three independent experiments are shown. VE-822- or PF-477736 treatment at the indicated dosages induced substantial viability reduction within 48 hours of drug exposure in four human SCLC cell lines (H-526, H-82, N-417, H-69). (**D–F**) Mice bearing xenograft tumors derived from three human SCLC cell lines (H-82, H526, H-69) were treated with VE-822, PF-477736 or vehicle solution. Tumor volumes were assessed by longitudinal caliper measurements every second day following treatment initiation. All vehicle-treated xenograft tumors displayed rapid growth. VE-822 and PF-477736 treatment significantly repressed tumor growth. (**I**) Immunoblot experiments using antibodies detecting total CHK1 (top panel), p-Ser-345-CHK1 (middle panel) and β-actin, which served as a loading control. Lysates derived from SCLC cell lines are loaded in the left lanes, while NSCLC lysates are loaded on the right side.

To further substantiate the validity of cell cycle checkpoint abrogation as a viable therapeutic concept for the treatment of SCLC, we next examined CHK1 mRNA expression levels in a collection of 970 human cancer cell lines from the COSMIC cell line project^43^, including 61 SCLC, 109 NSCLC and 800 non-lung cancer cell lines. In line with our data derived from primary tumor specimens (Fig. 1), we found that SCLC cell lines display significantly higher levels of *CHEK1* expression, than NSCLC and non-lung cancer cell lines (Fig. 6A). To assess whether the profound sensitivity to CHK1 inhibition that we had observed in murine cancer cell lines, was preserved in human SCLC cells, we next performed cell viability measurements in a panel of four human SCLC cell lines (H-526, H-82, N-417, H-69) and three human NSCLC lines (H-1975, HCC-44, A549). As shown in Figs 6B,C and S5A,B, VE-822 and PF-477736 induced substantially more viability reduction in human SCLC cells, compared to human NSCLC cells, thus confirming our observations in the murine system (cf. Figs 2, 3 and 4). Structure-specific off-target effects were excluded by replacing VE-822 and PF-477736 with alternative ATR- and CHK1 inhibitors that were based on entirely distinct chemical motifs. As shown in Fig. S5C,D, both inhibitors displayed similar cytotoxicity as VE-822 and PF-477736 in human SCLC cell lines. The *RB1* and *TP53* mutation status, as well as the copy number status of *MYC*, *MYCN* and *CCND1*, as curated in the Cancer Cell Line Encyclopedia (CCLE) database are indicated in Fig. S5E for H526, H82, H69, A549, H1975 and HCC44 cells^44^. H526, H69 and H82 cells are reported to carry bi-allelic *TP53* and *RB1* mutations. Intriguingly, a re-analysis of human lung cancer cell lines curated in the Catalogue Of Somatic Mutations in Cancer (COSMIC) database revealed that bi-allelic *TP53* and *RB1* aberrations can also be detected in a subset NSCLC lines (Fig. S5F).

To further probe the sensitivity of human SCLC cells against ATR- and CHK1 inhibitors, we next conducted therapy studies in xenograft tumors (Fig. 6D–F). For that purpose, we transplanted 1.5 × 10^6^ H-82 (Fig. 6D), H-526 (Fig. 6E) and H-69 (Fig. 6F) cells into the flanks of 8-week-old athymic NMRI-*Foxn1* ^*nu/nu*^ mice and allowed xenograft tumors to form. Tumor volumes were assessed by serial caliper measurements (Fig. S5G). Once, tumors reached a volume of 100mm^3^, treatment with VE-822 (30 mg/kg, orally, on days 1–3 of a 7-day cycle), PF-477736 (20 mg/kg, i.p., on days 1–5 of a 7-day cycle) or vehicle solution was initiated. While vehicle-treated xenograft tumors derived from H-82 (Fig. 6D), H-526 (Fig. 6E) and H-69 (Fig. 6F) cells displayed a rapid growth, both VE-822 and PF-477736 treatment led to a massively delayed tumor growth in all three models, which was mimicked by a substantial reduction in Ki67 staining on histological examination in tumors that were excised following completion of treatment (Fig. 6G,H). In line with this sensitivity of human SCLC xenografts, we found a substantially increased expression of CHK1 in SCLC cell lines, compared to NSCLC cells (Fig. 6I). Moreover, basal CHK1 activity, as evidenced by Ser-345 phosphorylation, was also increased in SCLC cells, compared to NSCLC controls (Fig. 6I). Thus, in summary, we demonstrate that murine and human SCLC cells and tumors display an actionable molecular dependence on the cell cycle checkpoint kinases ATR and CHK1, which could not be detected in murine and human NSCLC cells and tumors.

## Discussion

SCLC is a highly aggressive disease, which accounts for ~14% of all lung cancers^45^. Despite the conduction of a series clinical trials, including more than 40 phase III trials throughout the last 45 years, the principles of systemic treatment of SCLC patients with extensive stage disease have not substantially evolved over the past several decades^45^. This lack of therapeutic progress has left the 5-year overall survival rate below 7%^45^. This is in marked contrast to NSCLC, particularly adenocarcinoma, where genome sequencing efforts have revealed the identity of numerous recurrent, actionable somatic alterations, including oncogenic *EGFR* mutations, as well as *ALK*- and *ROS1* rearrangements^46^.

In the Western world, the first line treatment of SCLC patients presenting with extensive stage disease consists of a platinum-based combination chemotherapy, typically cis- or carboplatin in combination with etoposide^4,45^. In the relapsed setting, the topoisomerase-I poison topotecan is the only US Food and Drug Administration (FDA)-approved second line regimen for SCLC patients^45–47^. Further second line options beyond topotecan include taxanes, irinotecan, vinorelbine, and gemcitabine. In addition, temozolomide might be an attractive regimen for the second line situation, as it can be applied orally and displays activity in central nervous system lesions^48^. There is no internationally accepted consensus on systemic treatment regimens beyond first- and second-line therapy^45,49^. Furthermore, recently published exome and genome sequencing efforts have not revealed any recurrent actionable oncogenic genome aberrations^2,3^.

Here, we analyzed the transcriptomes of 214 human lung cancer patient samples, including 53 SCLC samples, as well as 970 human cancer cell lines, including 61 SCLC and 109 NSCLC cell lines. This analysis surprisingly revealed that SCLC tumors display a significantly higher expression of genes involved in cell cycle regulation, DNA damage signaling and DNA repair, than NSCLC tumors (Figs 1 and 6). One of the most strikingly differentially expressed genes was *CHEK1*. This *CHEK1* expression in human SCLC samples has recently also been reported in an independent study^24^. We further demonstrate that ATR- and CHK1 inhibitors display a selective toxicity in SCLC cells, as well as allograft and xenograft SCLC tumors, while NSCLC cell lines isolated from *Kras*-driven murine lung adenocarcinoma and allograft NSCLC tumors remained largely unaffected, when treated with these compounds (Figs 2, 3, 4 and 5). We note that it has not been demonstrated that either the re-expression of *TP53* and/or *RB1* in SCLC or the downregulation of these tumor suppressor genes in NSCLC may be specifically correlated with the CHK1- and ATR inhibitor response. Mechanistically, we show that exposure of SCLC cells leads to the accumulation of genotoxic damage and the induction of apoptotic cell death (Figs 2, 3 and 4). A possible explanation for the increased expression of cell cycle-regulating genes may lie in the uniform bi-allelic inactivation of *TP53* and *RB1* in SCLC^2,3^. This genomic constellation, which is a hallmark feature of SCLC^2,3^, likely promotes a substantial destabilization of cell cycle checkpoints in SCLC. While p53 transactivates a myriad of genes that repress cell cycle progression in response to genotoxic stress (e.g. *CDKN1A*, *14–3–3 s*, *GADD45A*, *RPRM* ^9^), RB1 governs the G_1_ restriction point through sequestration of E2F1^10–12^. Thus, combined loss of *TP53* and *RB1* is likely to promote unrestricted proliferation and a substantially impaired ability to halt the cell cycle in response to DNA damage. In line with a destabilized G_1_/S border as a common denominator for CHK1 inhibitor sensitivity, other groups have reported that *MYC*-driven diffuse large B cell lymphoma and *CCND1*-driven mantle cell lymphoma display a marked sensitivity against the CHK1 inhibitor PF-477736^50,51^. Altogether, it is tempting to speculate that SCLC cells display a compensatory upregulation of p53- and RB1-indpendent regulators of cell cycle progression, such as the checkpoint kinase CHK1. We note that a re-analysis of cell lines curated in the Catalogue Of Somatic Mutations In Cancer (COSMIC) database revealed that bi-allelic loss of *TP53* and *RB1* can also be detected in a subset of human NSCLC cancer cell lines (Fig. S5F). It would be interesting to assess the sensitivity of these cell lines against ATR- and CHK1 inhibitors.

Intriguingly, numerous checkpoint kinase inhibitors, including CHK1- and combined CHK1/CHK2 inhibitors have been developed and are currently evaluated in phase I and II clinical trials, either as single agents or in combination with chemotherapeutic compounds^52,53^. No clinically-relevant genotype-mapped drug response predictors have been reported for CHK1 inhibitors, to date. However, the CHK1 inhibitor prexasertib (LY-2606368) is currently undergoing evaluation as a single agent in a phase II trial with extensive stage SCLC. Given the data reported in our study, it will be interesting to see whether the results reported in the prexasertib trial will lend further support to the clinical development of CHK1 inhibitors for the treatment of SCLC patients. It has also been reported that ATR- and CHK1 inhibitors display synergistic activity in cancer cells^54^. It will thus be interesting to study whether combined inhibition of ATR and CHK1 may display selective synergistic activity in SCLC.

## Materials and Methods

### Gene expression analysis

Transcriptome sequencing data of human lung tumors was analyzed by referring to the RNA-seq data of 101 lung adenocarcinomas^25,27,28^, 60 lung squamous cell carcinoma samples^26^ and 53 SCLC tumor samples^2,3^. Gene-specific transcripts were quantified with RSEM^55^. Differentially expressed genes were determined with the SAM R package at a *Q*-value < 0.05^56^. Differentially expressed gene expression profiles were analyzed with DAVID^57^.

### Autochthonous models of small cell lung cancer and lung adenocarcinoma

To induce murine SCLC and lung adenocarcinoma, we applied 2.5 × 10^7^ PFU Adeno-CMV-Cre intratracheally to *Rb1* ^*fl/fl*^ *;Tp53* ^*fl/fl*^ (RP) and *Kras* ^*LSL.G12D/wt*^ *;Tp53* ^*fl/fl*^ (KP) mice, respectively^29,30^. 10-week-old animals were anesthetized with Ketavet (100 mg/kg) and Rompun (20 mg/kg) by intraperitoneal injection, as previously described^31,32,58^. RP mice were scanned by MRI under isoflurane (2.5%) anesthesia to confirm tumor formation approximately 30 weeks after Adeno-Cre application. KP mice were scanned by µCT imaging (Aloka, Latheta LCT-100) under isoflurane (2.5%) anesthesia to confirm tumor formation 5 weeks after Adeno-Cre application. Mice were kept on a *C57Bl6* background. The local authorities and the local animal protection committee approved all animal procedures. All mouse experiments were conducted in accordance with our Institutional Animal Care and Use Committee (IACUC). All animal experiments were approved by the responsible local government authorities, namely the Landesamt für Natur, Umwelt und Verbraucherschutz Nordrhein Westfalen. All animal experiments were carried out in accordance with the relevant guidelines and regulations.

### Allograft mouse model

For the allograft experiments, we anesthetized (2.5% isoflurane) and injected 8–12-week-old wildtype *C57Bl6*mice with 2 × 10^6^ murine SCLC tumor cells (RP1, 3 and 4) and 1.5 × 10^6^ murine NSCLC tumor cells (KP1 and 2) into the right lung. Tumor formation was verified by µCT imaging 9 days after injection. SCLC- and NSCLC tumor-bearing mice were randomly allocated to receive four cycles of VE-822, PF-477736 or vehicle, as indicated. Tumor volume changes were monitored by µCT imaging under 2.5% isoflurane at the end of two treatment cycles and assessed by OsiriX and DICOM viewer software packages (OsiriX v.8.2, Pixmeo, Switzerland). We normalized the tumor volumes under treatment to baseline tumor volumes, assessed prior to therapy initiation. Survival curves were calculated up to 175 days after the injection of tumor cells. The local authorities and the local animal protection committee approved all animal procedures. All mouse experiments were conducted in accordance with our Institutional Animal Care and Use Committee (IACUC). All animal experiments were approved by the responsible local government authorities, namely the Landesamt für Natur, Umwelt und Verbraucherschutz Nordrhein Westfalen. All animal experiments were carried out in accordance with the relevant guidelines and regulations.

### Xenograft mouse model

We generated 120 xenograft tumors from three different human SCLC cell lines (H-69, H-82, H-526). For each xenograft tumor, we collected 1.5 × 10^6^ tumor cells, re-suspended them in serum-free media and subcutaneously injected them into the flanks of 8-week-old male athymic nude mice (NMRI-*Foxn1* ^*nu/nu*^, Janvier). Mice were monitored for the formation of palpable subcutaneous tumors. Upon manifestation of tumors (≥100 mm³), VE-822 or PF-477736 were administered for two cycles following the protocol described above. Diameters of subcutaneous tumors were measured by a digital caliper every second day following initiation of therapy. For tumor volume measurements, mice were anesthetized with 2.5% isoflurane. Tumor volumes were calculated, based on the modified ellipsoid formula V = 1/2 (width × length²). We normalized the tumor volumes under treatment to baseline tumor volumes, assessed prior to therapy initiation. In accordance with our Institutional Animal Care and Use Committee (IACUC), mice with a single tumor volume larger than 1.500 mm³ were sacrificed. The local authorities and the local animal protection committee approved all animal procedures. All mouse experiments were conducted in accordance with our Institutional Animal Care and Use Committee (IACUC). All animal experiments were approved by the responsible local government authorities, namely the Landesamt für Natur, Umwelt und Verbraucherschutz Nordrhein Westfalen. All animal experiments were carried out in accordance with the relevant guidelines and regulations.

### Histological analysis

Mice harboring SCLC or NSCLC tumors were sacrificed at the indicated time points and lungs were fixed in 4% PFA. Formalin-fixed paraffin-embedded (FFPE) murine lung samples were cut into 4 μm thick sections and mounted on slides. After staining with haematoxylin and eosin (H&E) or Ki67 the samples were assessed by two independent board-certified pathologists (M.W., R.B.).

### Cell lines and cell culture

Individual murine SCLC and NSCLC tumor nodules derived from individual RP and KP mice, respectively, were isolated and cultured in RPMI medium supplemented with 1% penicillin/streptomycin and 10% fetal calf serum, to derive stable tumor cell lines, as previously described^31,32,58^. Human cell lines were obtained from the American Type Culture Collection (ATCC), the German Resource Center for Biological Material (DSMZ), or were a kind gift from Roman K. Thomas (Department of Translational Genomics, University of Cologne). All our cell lines were cultured at 37 °C in a humidified incubator supplemented with 5% CO_2_. RPMI medium supplemented with 10% fetal calf serum (FCS) and 1% penicillin/streptomycin was the culture medium used for all cell lines. We passaged all adherent cells lines by washing with PBS and subsequent incubation in trypsin/EDTA. By addition of culture medium, we inactivated trypsin and plated or diluted the cells afterwards. Passaging of our suspension cell lines was executed by suitable dilution of the cell suspension and adding fresh culture medium. All cell lines were routinely tested for infection with mycoplasma (MycoAlert, Lonza).

### Population doubling

To assess population doubling times, triplicates of each of the five murine SCLC cell lines and the five murine NSCLC cell lines were seeded in 10 mm dishes at a density of 1 × 10^6^ cells per dish for SCLC and 750,000 cells per dish for NSCLC. After 48 hours, cells were trypsinized and counted on a Beckman Coulter cell counter. Subsequently, cells were seeded at the above-mentioned density. The experiment was performed over 12 days, resulting in a total of 7 passages. Doubling times were calculated using the following equation: PD = t × Log2/(LogC2-LogC1), where PD = Population doubling; t = time (hours); Log = 10 based log; C1 = first count; C2 second count.

### Chemicals

Cisplatin was purchased from Accord Pharmaceuticals and etoposide was from Hexal Pharma. VE-822 and PF-477736, as well as AZD-7762 were purchased from Abmole and AZD-6738 was from Selleckchem. All small molecule compounds were dissolved in DMSO and stored at −80 °C or −20 °C, according to instructions.

### Preparation of compound solutions for in vivo application

The ATR inhibitor VE-822 (Abmole, M3115) was dissolved in an equilibrated (v/v) mixture of PBS (70%), Polyethylene glycol 300 (PEG300, 29.5%) and Tween 80 (0.5%) at a concentration of 18.0 mg/ml. The Chk1 inhibitor PF-477736 (Abmole, M1764) was prepared in a 1:1 (v/v) mixture of PBS and dimethyl sulfoxide at a concentration of 10.0 mg/ml. The following treatment regimens were applied in the allograft and the xenograft mouse model:

- Control cohort: daily intraperitoneal injection of PBS/DMSO vehicle solution.
- VE-822 cohort: weekly for three consecutive days at a dose of 30 mg/kg by oral gavage.
- PF-477736 cohort: weekly for five consecutive days at a dose of 20 mg/kg by intraperitoneal injection.

### Cell viability assay

To assess cell viability, we employed CellTiterGlo^®^ (Promega) assays, according to the instructions provided by the manufacturer. In brief, 100 µl of each cell line were plated into 96-well plates at a density of 5.000 cells per well. 24 hours after seeding, triplets of each cell line were treated, as indicated. Each drug concentration was pre-prepared in deep-well plates. 25 µl of this stock solution was transferred from the deep-well plate to the 96-well plates. Cells were exposed to treatment for 48 hours in a humidified incubator at 37 °C and 5% CO_2_. After incubation, 125 µl of the CellTiterGlo^®^ reagent were added to each well of the 96-well plate. The contents were mixed for 2 minutes in an orbital shaker to induce cell lysis. After a 10-minute incubation at room temperature, relative cell viability was determined by measuring the ATP content in each well on a luminescence plate reader, equipped with an automated stacking module (Tecan). We normalized luminescence values to luminescence intensities of control wells on the same plate, treated with vehicle solution.

### Flow cytometry

In the flow cytometry experiments reported here, we used Annexin-V/PI staining to quantify the induction of apoptosis, exactly as previously described^59,60^. All murine SCLC and NSCLC cell lines were seeded into 6-well plates at approximately 50% density and were incubated for 24 hours in a humidified incubator at 37 °C and 5% CO_2_. Subsequently, triplets of each cell line were either left untreated or were exposed to one of the following inhibitors and dosages: cisplatin (0.1 µM, 2.5 µM, 10.0 µM), etoposide (1.0 µM, 5.0 µM and 20.0 µM), VE-822 (0.1 µM, 0.75 µM, 3.0 µM) or PF-477736 (0.1 µM, 1.0 µM, 3.0 µM). After 48 hours of drug exposure, cells were trypsinized and harvested. After washing them twice in cold PBS, cells were re-suspended in an antibody-binding buffer (0.1 M HEPES [pH 7.4], 140 mM NaCl; 25 mM CaCl_2_) and incubated for 15 minutes in Annexin-V (BD 556420) plus propidium iodide (PI) (Carl Roth, CN74, 0,5 mg/ml in NaCl). Annexin-V/PI staining intensity was subsequently assessed by flow cytometry (Gallios, Beckman Coulter). A minimum of 100,000 events was analyzed for each measurement and apoptotic cells were defined as double-positive.

### Immunofluorescence

Murine SCLC and NSCLC cells were seeded at a confluency of approximately 50% on 6-well plates with a 22-mm glass coverslip on the bottom. The cells were treated with VE-822 (0.75 µM) or PF-477736 (1.0 µM) or were left untreated for 12, 24 or 48 hours. Cells were fixed in 4% PFA (15 minutes on ice) and the coverslips were subsequently washed in PBS. Cells were subsequently permeabilized on ice for 10 minutes in cytoskeleton buffer (100 mM piperazine-N,N′-bis[2-ethanesulfonic acid], PIPES [pH 6.8], 100 mM NaCl, 300 mM sucrose, 3 mM MgCl_2_, 1 mM EDTA, 0.5% Triton-X100) and incubated with cytoskeleton stripping buffer (10 mM Tris-HCl [pH7.4], 10 mM NaCl, 3 mM MgCl_2_, 2% Tween 20, 0.5% sodium deoxycholate) under the same conditions. Samples were then blocked in 5% bovine serum albumin (BSA); 2% normal goat serum (NGS), 0.01% Triton X100 in PBS and incubated overnight at 4 °C with a primary antibody detecting γH2AX (Abcam ab22551, 1:250). After 18 hours of incubation, we executed a 1 hour incubation with a secondary antibody goat-anti-mouse Alexa488 (Invitrogen, A21121, 1:600) followed by several washes. Finally, we mounted cells with ProLong Gold antifade reagent with DAPI (Life Technology, P36935), analyzed them through microscopy, using an AxioImager M1 (Carl Zeiss) and counted the γH2AX-positive cells.

### Immunoblotting

Cells were treated as indicated and subsequently washed with PBS, lysed in RIPA lysis buffer (20 mM Tris-HCl [pH 7.5], 150 mM NaCl, 1 mM Na2EDTA, 1 mM EGTA, 1% NP-40, 1% sodium deoxycholate, 2.5 mM sodium pyrophosphate, 1 mM beta-glycerolphosphate, 1 mM Na3VO4, 1 µg/ml leupeptin) and lysates were supplemented with 1% SDS and 100 μM PMSF. Samples were subsequently sonicated for 10 seconds and centrifuged at 12.500 g for 30 minutes. We transferred the supernatants into fresh tubes and examined the protein concentration with a BSA standard curve, using a Bradford assay (Thermo Scientific). Equivalent amounts of protein (up to 90 µg) were boiled at 95 °C for 5 minutes in Laemmli sample buffer, separated using 10% SDS-PAGE and blotted on PVDF membranes (Immobilon-FL, IPFL00010, Merck Millipore, 0.45 μm pore size). Membranes were blocked in 5% BSA in TBS-T for 1 hour and stained with specific antibodies. The γH2AX antibody (Millipore, 05–636, diluted 1:1.000) was applied overnight. The β-actin antibody (A5316, Sigma Aldrich, diluted 1:10.000) was applied for 20 minutes. Secondary antibody staining was performed following several washing steps in TBS-T. The secondary antibody was applied for 3 hours. After several washing steps (3X TBS-T, 2x PBS), we imaged the membranes on a digital ChemiDoc XRS+ System (BioRad), using the Amersham ECL Western Blotting Detection Reagent (GE Healthcare) and analyzed them with the manufacturer’s software (Image Lab, BioRad). γH2AX expression relative to the vehicle-treated control was analyzed through densitometry, using ImageJ version 1.51 h. Original full-length immunoblot membranes are shown in Figs S6 and S7.

### Statistical analysis

Statistical analysis was performed using GraphPad Prism Version 5.00 (GraphPad Software, La Jolla, CA) and Excel (Microsoft, Redmond, WA). We used student’s two-tailed t-test, log-rank (Mantel-Cox) test and one-way-Anova test to determine statistical significance. Significance was considered on p-values below 0.05. Levels of significance were *p < 0.05; **p < 0.01; ***p < 0.001. Errors bars in figures represent standard deviation, if not differently indicated.

### Data Availability Statement

The datasets generated during and/or analyzed during the current study are available from the corresponding author on reasonable request.

## Electronic supplementary material

Supplementary Information
