# Supplementary material for: Targeting a non-oncogene addiction to the ATR/CHK1 axis for the treatment of small cell lung cancer
Source: Sci Rep. 2017 Nov 14;7:15511. doi: 10.1038/s41598-017-15840-5 (PMC5686113; doi:10.1038/s41598-017-15840-5)
Supplement: Supplementary file 1 — Supplementary Information [file 41598_2017_15840_MOESM1_ESM.pdf]

Supplementary Material

Targeting a non-oncogene addiction to the ATR/CHK1 axis for the treatment of small cell lung cancer

Fabian Doerr[1,2,3], Julie George[4], Anna Schmitt[1,2], Filippo Beleggia[1,2], Tim Rehkämper[1,2], Sarah Vierkotten[1,2], Vonn Walter[5,6], Jean-Philip Weber[7], Roman K. Thomas[4,8,9], Maike Wittersheim[8], Reinhard Büttner[8], Thorsten Persigehl[7], H. Christian Reinhardt[1,2]

[1] Department I of Internal Medicine, University Hospital of Cologne, Cologne, Germany.
[2] Cologne Excellence Cluster on Cellular Stress Response in Aging-Associated Diseases, University of Cologne, Cologne, Germany.
[3] Department of Cardiothoracic Surgery, University Hospital of Cologne, Cologne, Germany.
[4] Department of Translational Genomics, Center of Integrated Oncology Cologne-Bonn, Medical Faculty, University of Cologne, Cologne, Germany.
[5] Department of Public Health Sciences, Penn State Milton S. Hershey Medical Center, Hershey, PA, USA
[6] Lineberger Comprehensive Cancer Center, University of North Carolina at Chapel Hill, Chapel Hill, NC, USA.
[7] Department of Radiology, University Hospital of Cologne, Cologne, Germany.
[8] Institute for Pathology, University Hospital of Cologne, Cologne, Germany.
[9] German Cancer Research Center, German Cancer Consortium (DKTK), Heidelberg, Germany.

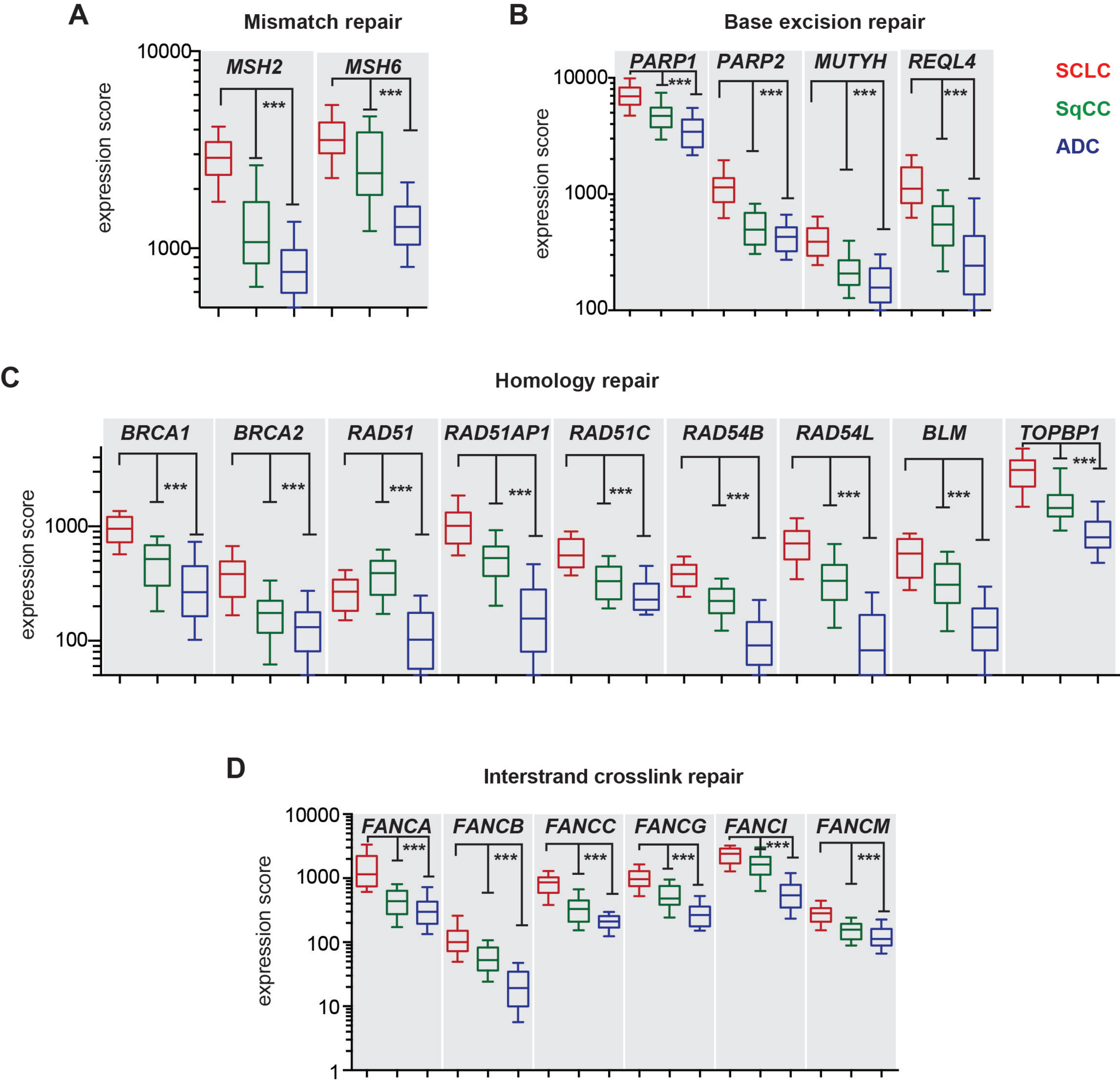
A
Mismatch repair
MSH2
MSH6
***
expression score
10000
1000
B
Base excision repair
PARP1
PARP2
MUTYH
REQL4
10000
1000
100
SCLC
SqCC
ADC
C
Homology repair
BRCA1
BRCA2
RAD51
RAD51AP1
RAD51C
RAD54B
RAD54L
BLM
TOPBP1
100
D
Interstrand crosslink repair
FANCA
FANCB
FANCC
FANCG
FANCI
FANCM
10
1

Supplementary Figure 1: SCLC displays increased mRNA expression levels of numerous genes involved in different DNA repair pathways, compared to SqCC and ADC.

Displayed are the expression levels of different genes involved in (**A**) mismatch repair (*MSH2*, *MSH6*), (**B**) base excision repair (*PARP1*, *PARP2*, *MUTYH*, *REQL4*), (**C**) homology-directed repair (*BRCA1*, *BRCA2*, *RAD51*, *RAD51AP1*, *RAD51C*, *RAD54B*, *RAD54L*, *BLM*, *TOPBP1*) and (**D**) interstrand crosslink repair (*FANCA*, *FANCB*, *FANCC*, *FANCG*, *FANCI*, *FANCM*). Expression levels are displayed as a box plots. Whiskers indicate the 10-90 percentile. ***<0.0001 (Mann Whitney test). The histological annotation of the lung tumor samples is provided in the color panel (top right).

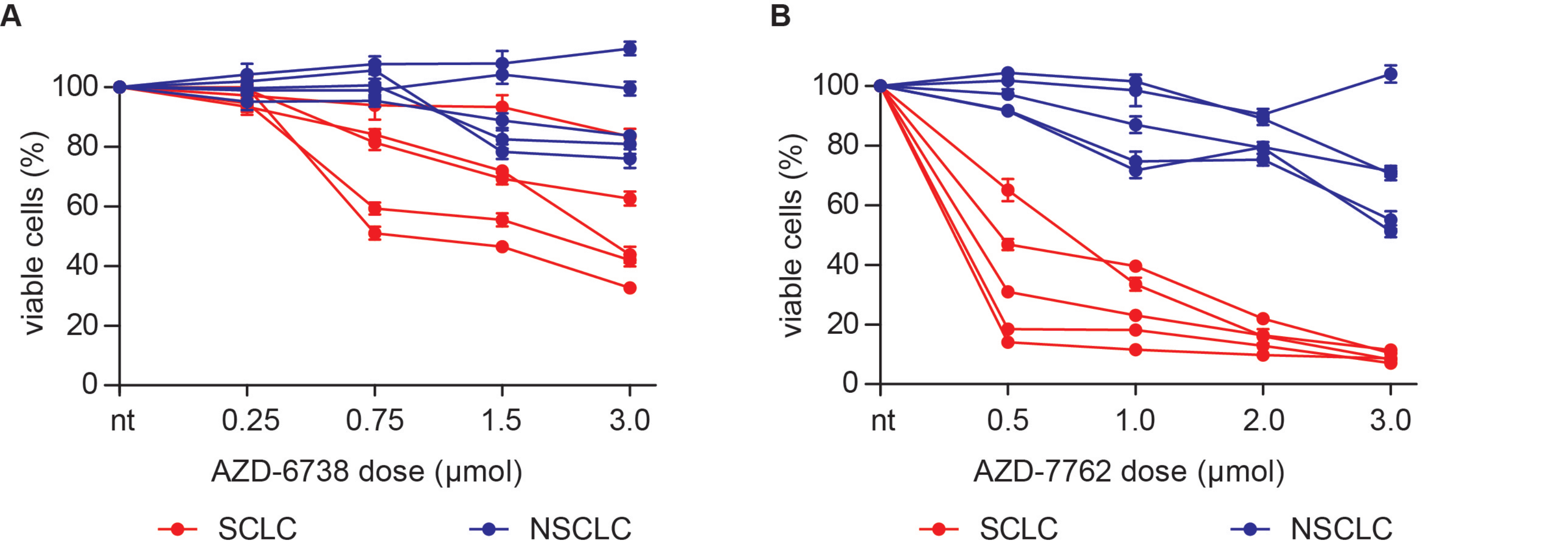
A
viable cells (%)
100
80
60
40
20
0
nt
0.25
0.75
1.5
3.0
AZD-6738 dose (µmol)
SCLC
NSCLC
B
viable cells (%)
100
80
60
40
20
0
nt
0.5
1.0
2.0
3.0
AZD-7762 dose (µmol)
SCLC
NSCLC

Supplementary Figure 2: Alternative ATR- and CHK1 inhibitors display similar cytotoxicity, as VE-822 and PF-477736 in SCLC cell lines.

(**A-B**) Intracellular ATP was measured to assess cell viability. Average values of three independent experiments are shown. RP cell lines were more sensitive to ATR inhibition (AZD-6738) and CHK1 inhibition (AZD-7762), than KP cell lines.

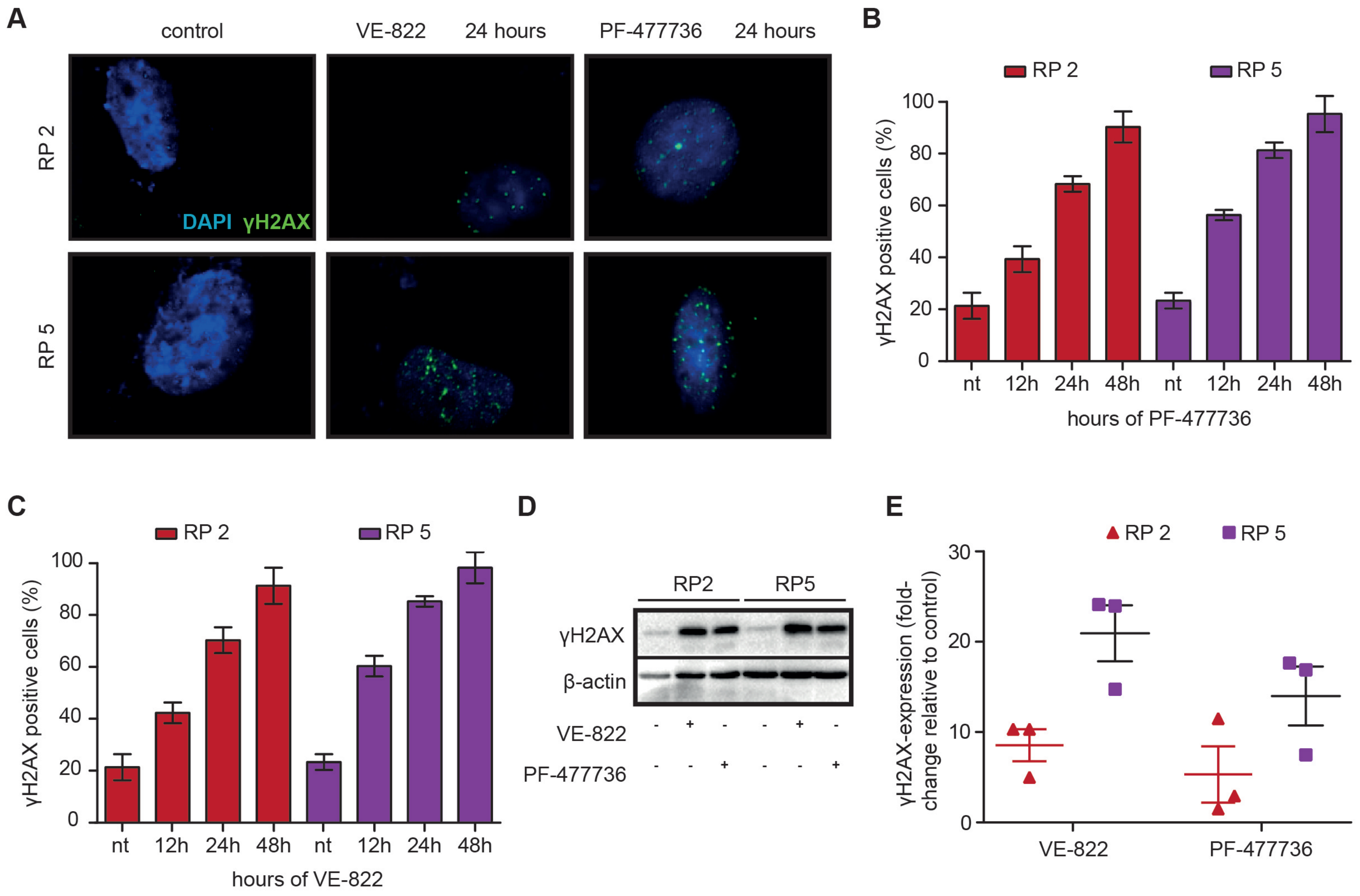
A
control
VE-822 24 hours
PF-477736 24 hours
RP 2
RP 5
DAPI γH2AX
B
RP 2
RP 5
γH2AX positive cells (%)
100
80
60
40
20
0
nt 12h 24h 48h nt 12h 24h 48h
hours of PF-477736
C
RP 2
RP 5
γH2AX positive cells (%)
100
80
60
40
20
0
nt 12h 24h 48h nt 12h 24h 48h
hours of VE-822
D
RP2
RP5
γH2AX
β-actin
VE-822
PF-477736
E
RP 2
RP 5
γH2AX-expression (fold-change relative to control)
30
20
10
0
VE-822
PF-477736

Supplementary Figure 3: Genotoxic damage in murine SCLC cell lines following ATR- and CHK1 inhibition is independent of growth kinetics.

(**A-C**) RP 2 and 5 cell lines were stained with an antibody detecting γH2AX and a DAPI counterstain. VE-822 and PF-477736 induced genotoxic damage in RP2 and RP5 cells.

(**A**) Representative immunofluorescence images are shown for RP 2 and 5 cell lines following a 24-hour exposure to the indicated treatments.

(**B-C**) The percentage of γH2AX-positive cells was quantified. VE-822 (0.75μM) and PF-477736 (1.0μM) induced DNA damage after a drug exposure of 12, 24 and 48 hours in both RP cell lines.

(**D-E**) Immunoblot was executed on RP 2 and 5 cell lines after VE-822 (0.75μM) or PF-477736 (1μM) treatment for 48 hours. Vehicle-treated cells served as a control. β-actin was used as a loading control.

(**D**) Representative immunoblot images are shown for RP 2 and 5 cells.

(**E**) The intensity of enhanced chemiluminescence signal was quantified using densitometry. RP 5 cells displayed a stronger γH2AX signal, than RP 2 cells following VE-822 and PF-477736 treatment.

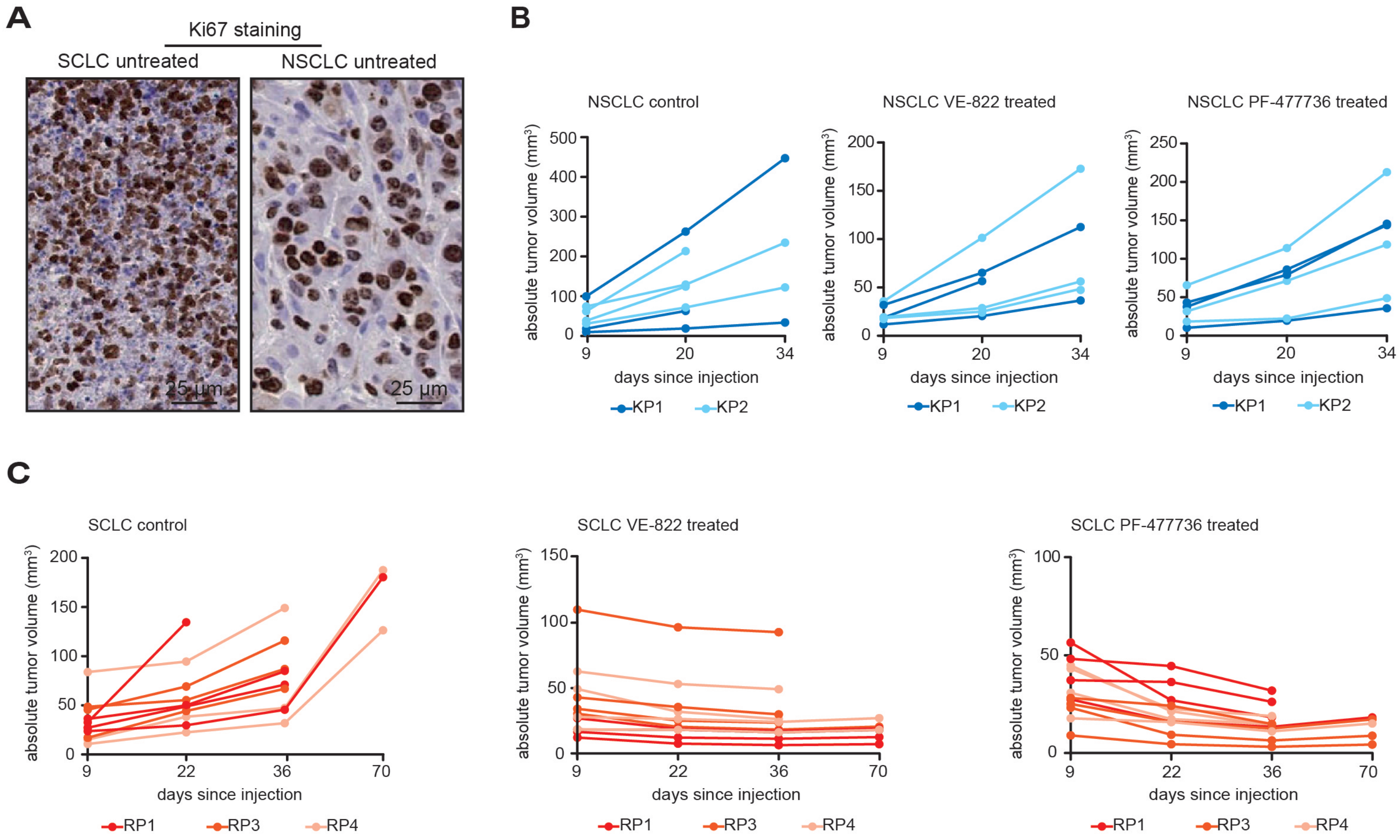

A
Ki67 staining
SCLC untreated
NSCLC untreated
25 µm
25 µm
B
NSCLC control
NSCLC VE-822 treated
NSCLC PF-477736 treated
absolute tumor volume (mm3)
days since injection
KP1
KP2
C
SCLC control
SCLC VE-822 treated
SCLC PF-477736 treated
absolute tumor volume (mm3)
days since injection
RP1
RP3
RP4

Supplementary Figure 4: ATR- and CHK1 inhibition is therapeutically effective in SCLC tumors, *in vivo*.

(**A**) Representative images of Ki67 staining form untreated mice bearing SCLC or NSCLC tumors from the allograft model. Scale bar 25µm.

(**B**) Absolute tumor volume is displayed in mm^3 for all NSCLC-bearing mice in the vehicle-treated cohort (n=7) and in the VE-822- (n=6) and PF-477736-treated (n=6) cohort. Three animals in the vehicle-treated cohort and one animal in the VE-822-treated cohort died before completion of cycle 4.

(**C**) Absolute tumor volume is displayed in mm^3 for all SCLC bearing mice in the vehicle-treated cohort (n=10) and in the VE-822- (n=12) and PF-477736-treated (n=12) cohort. One animal in the vehicle-treated cohort died before completion of cycle 4. The post treatment phase until day 70 was survived by 15 animals (vehicle-treated n=3; VE-822-treated n=7, PF-477736-treated n=5).

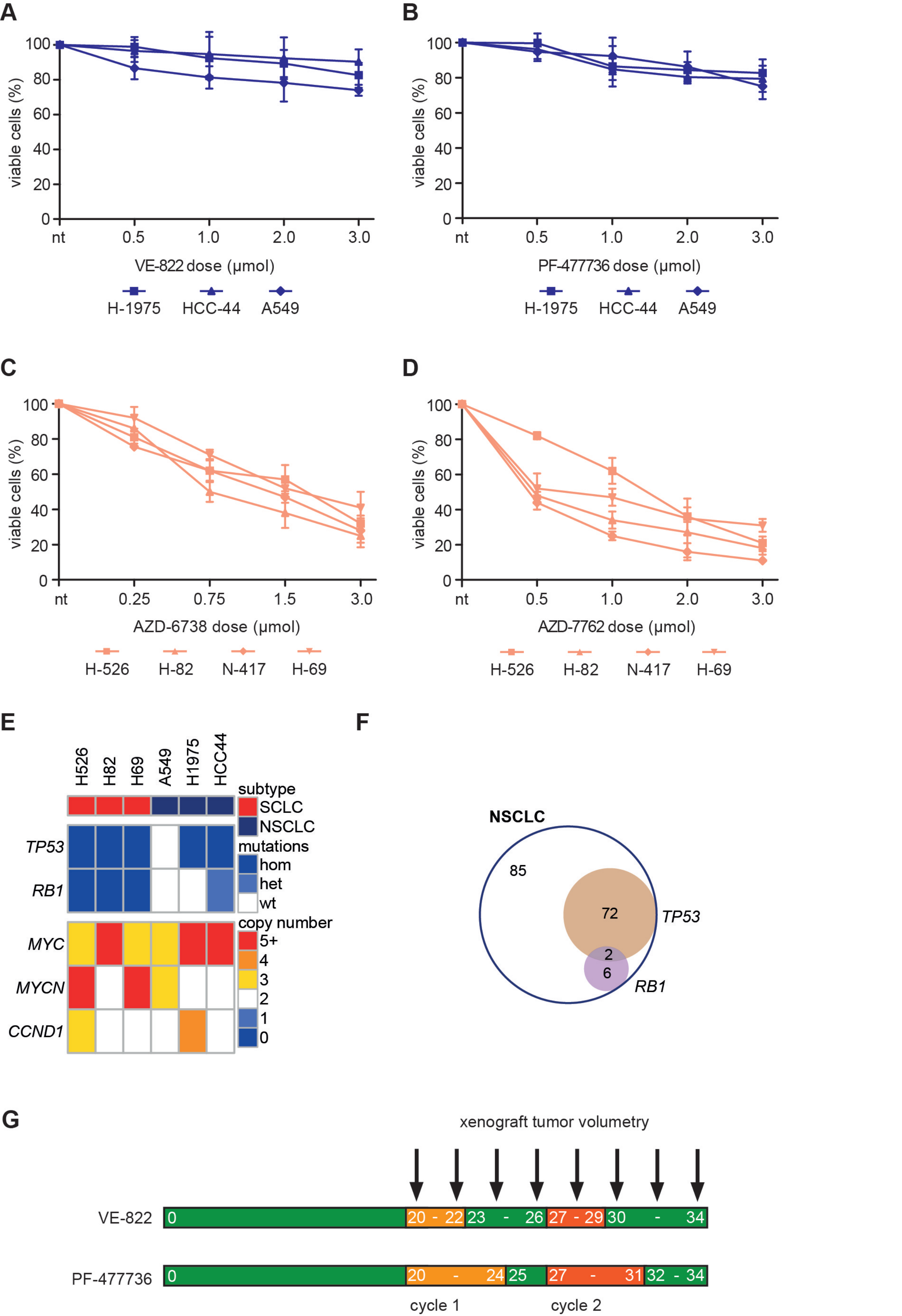

A
viable cells (%)
100
80
60
40
20
0
nt
0.5
1.0
2.0
3.0
VE-822 dose (µmol)
H-1975
HCC-44
A549
B
viable cells (%)
PF-477736 dose (µmol)
H-1975
HCC-44
A549
C
viable cells (%)
nt
0.25
0.75
1.5
3.0
AZD-6738 dose (µmol)
H-526
H-82
N-417
H-69
D
viable cells (%)
AZD-7762 dose (µmol)
H-526
H-82
N-417
H-69
E
H526
H82
H69
A549
H1975
HCC44
subtype
SCLC
NSCLC
TP53
RB1
mutations
hom
het
wt
MYC
MYCN
CCND1
copy number
5+
4
3
2
1
0
F
NSCLC
85
72
TP53
2
6
RB1
G
xenograft tumor volumetry
VE-822
0
20 - 22
23 - 26
27 - 29
30 - 34
PF-477736
0
20 - 24
25
27 - 31
32 - 34
cycle 1
cycle 2

Supplementary Figure 5: VE-822 and PF-477736 induce substantially more viability reduction in human SCLC cells, compared to human NSCLC cells.

(**A-D**) Intracellular ATP was measured to assess cell viability. Average values of three independent experiments are shown.

(**A-B**) Three different human NSCLC cell lines (H-1975, HCC-44, A549) were treated with the indicated dosages of VE-822 or PF-477736 over 48 hours. Only a mild viability reduction was observed after both treatments.

(**C-D**) Human SCLC cell lines (H-526, H-82, N-417, H-69) are sensitive to ATR inhibition (AZD-6738) and CHK1 inhibition (AZD-7762).

(**E**) *TP53*- and *RB1* mutation status, as well as *MYC*, *MYCN* and *CCND1* copy number status of H526, H82, H69, A549, H1975 and HCC44 cells. Data are derived from the Catalogue Of Somatic Mutations In Cancer (COSMIC) database.

(**F**) Re-analysis of TP53 and *RB1* mutation data derived from human NSCLC cell lines curated in the COSMIC database. Mutation frequency is represented as a Venn diagram.

(**G**) Three different human SCLC cell lines (H-526, H-82, H-69) were transplanted into the flanks of athymic mice. For each cell line mice were allocated to a control cohort (not shown in E) and two treatment cohorts. Two cycles of treatment were applied. The VE-822 treated cohort received treatment weekly for three consecutive days at a dose of 30mg/kg by oral gavage. The PF-477736 treated cohort received treatment weekly for five consecutive days at a dose of 20mg/kg by intraperitoneal injection. Tumor volumes were assessed by serial digital caliper measurements.

Full length membranes related to Figure 3C

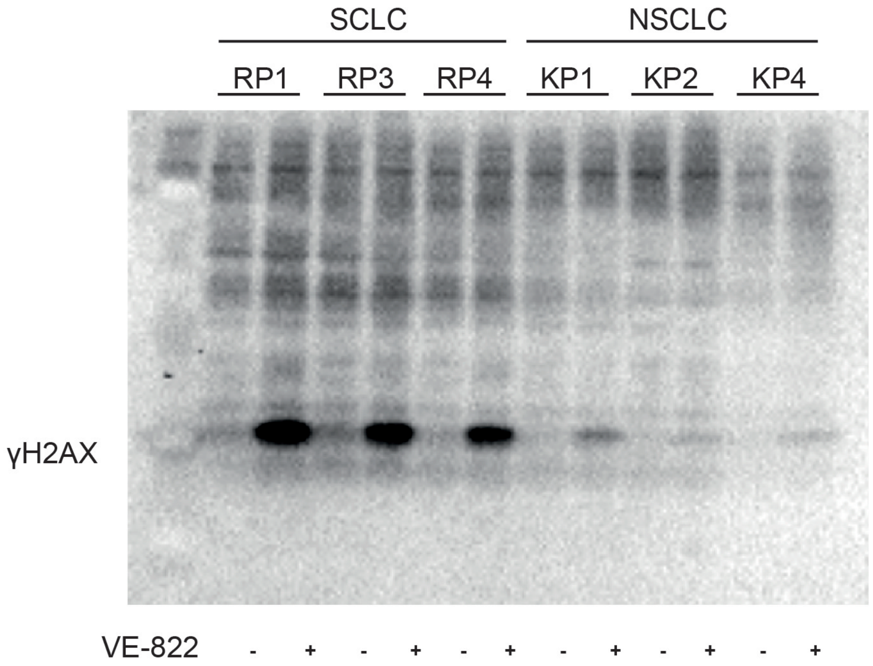

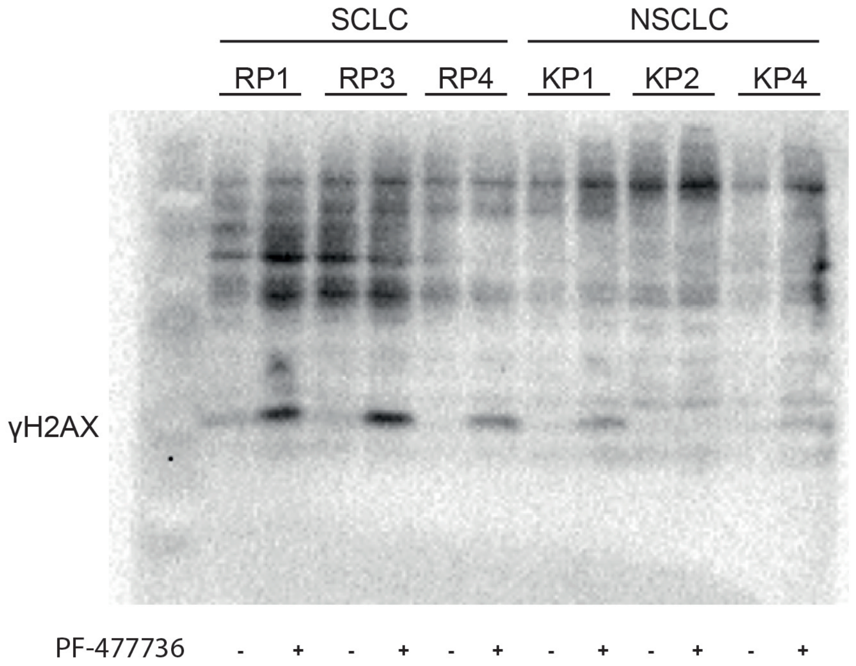

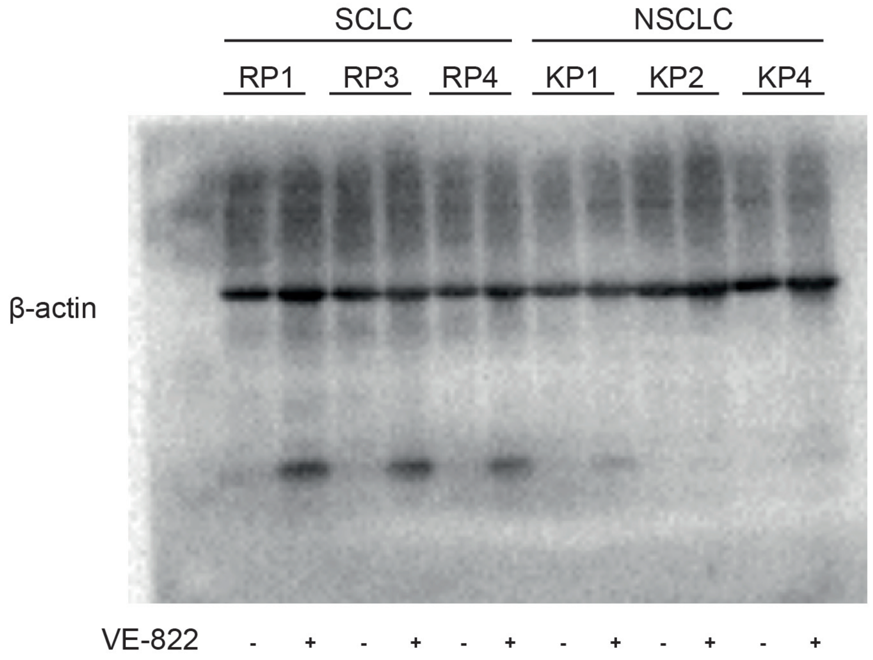

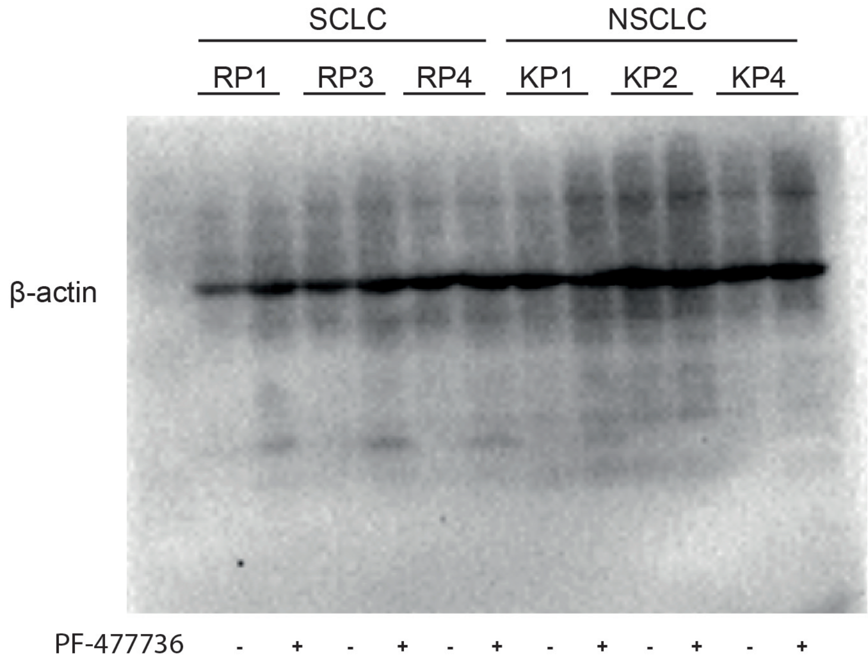

Full length membranes related to Supplemental Figure 3D

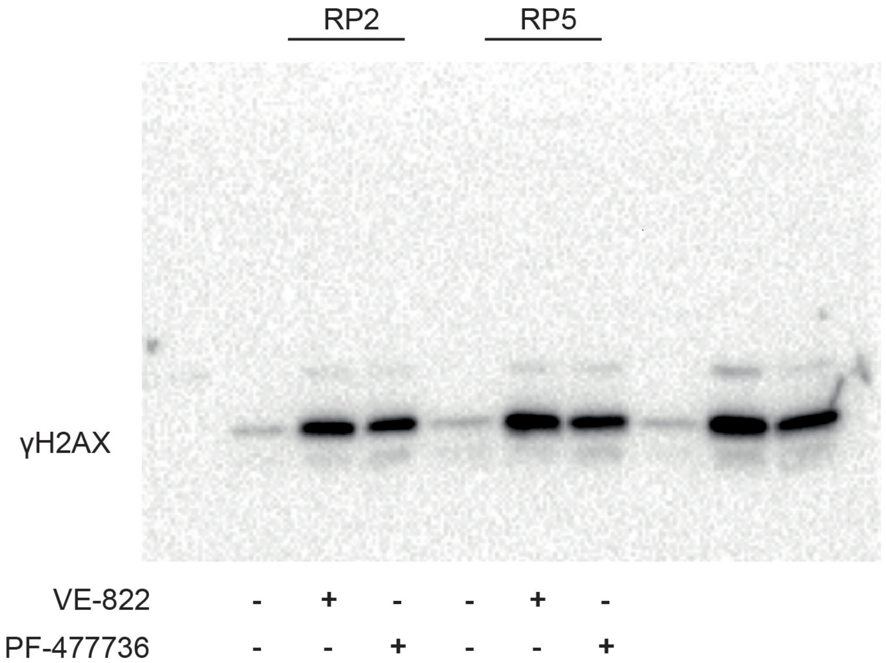

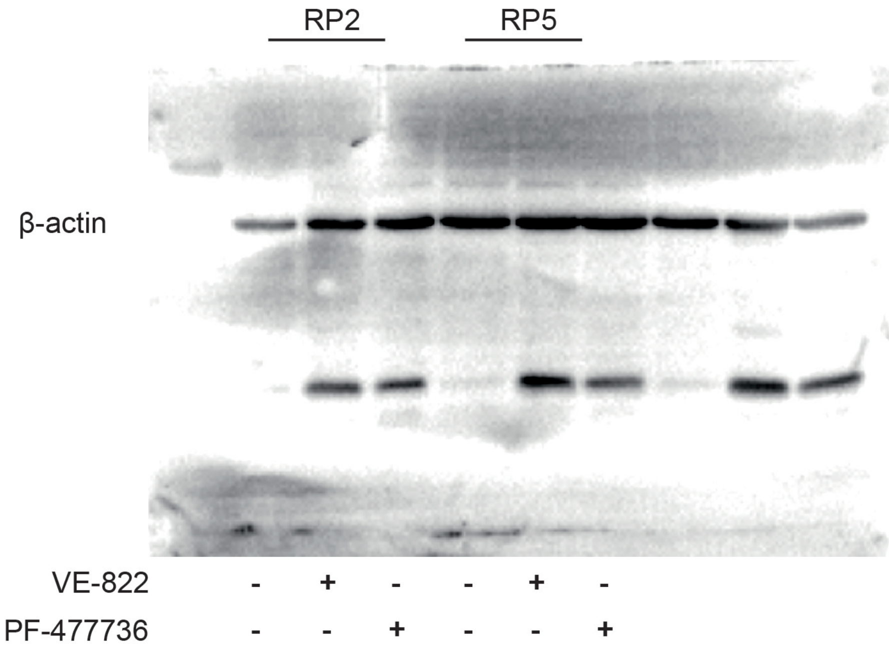

Supplementary Figure 6: Original full-length blots related to Figure 3.

The original full-length immunoblots related to Figure 3C (top) and Supplemental Figure 3D are shown.

Full length membranes related to Figure 6I

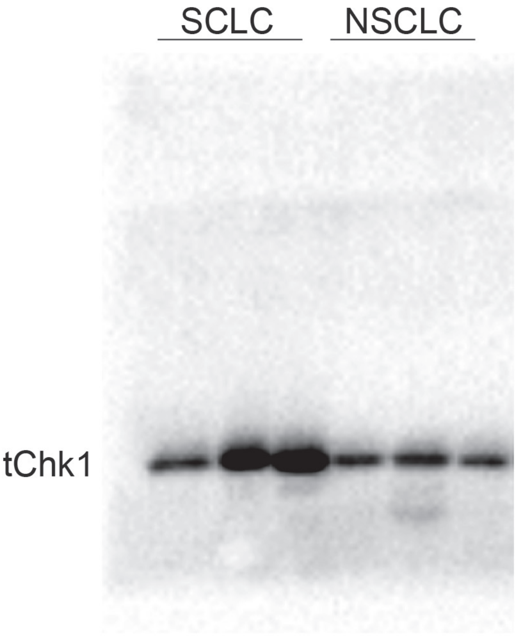

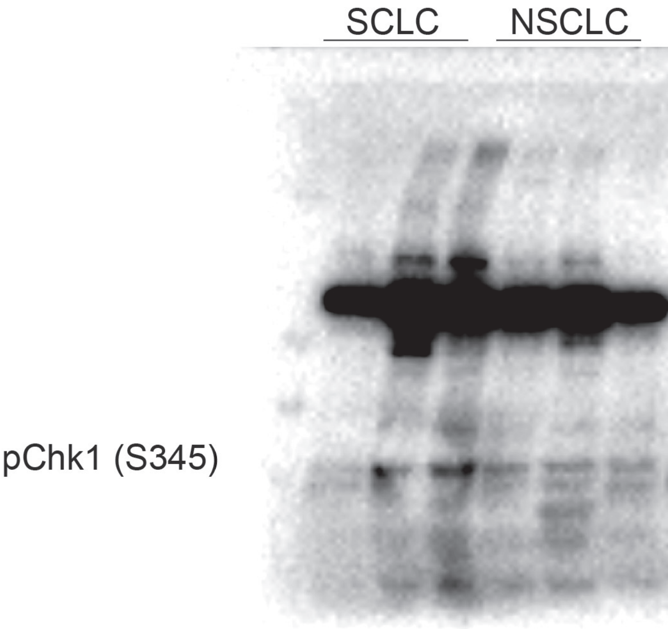

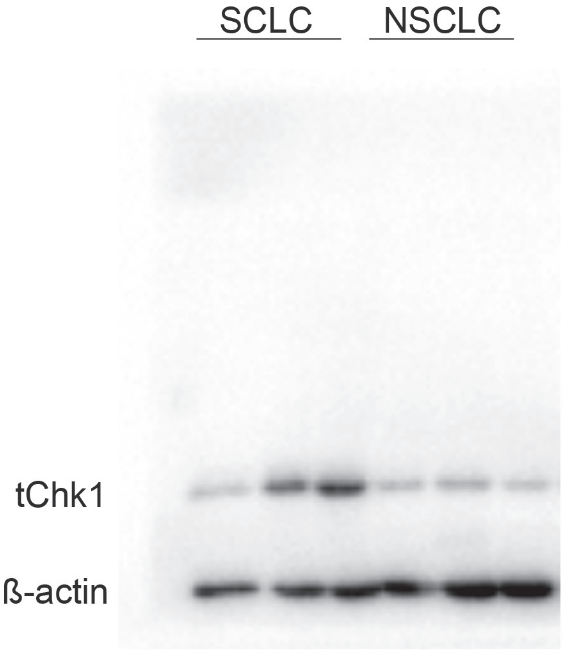

Supplementary Figure 7: Original full-length blots related to Figure 6.

The original full-length immunoblots related to Figure 6I are shown.
